# Analyzing a faculty online learning community as a mechanism for supporting faculty implementation of a guided-inquiry curriculum

**DOI:** 10.1186/s40594-020-00268-7

**Published:** 2021-02-24

**Authors:** Edward Price, Alexandra C. Lau, Fred Goldberg, Chandra Turpen, P. Sean Smith, Melissa Dancy, Steve Robinson

**Affiliations:** 1 Department of Physics, California State University San Marcos, 333 South Twin Oaks Valley Road, San Marcos, CA 92096 USA; 2 Western Michigan University, Center for Research on Instructional Change in Postsecondary Education, Kalamazoo, MI 49008 USA; 3 San Diego State University, Center for Research in Mathematics and Science Education, 6475 Alvarado Road, Suite 206, San Diego, CA 92120 USA; 4 Department of Physics, University of Maryland, 1312 Toll Physics Building, College Park, MD 20742 USA; 5 Horizon Research, Inc., 326 Cloister Court, Chapel Hill, NC 27514 USA; 6 Department of Physics, University of Colorado, 390 UCB, Boulder, CO 80309 USA; 7 Department of Physics, Tennessee Technological University, Box 5051, Cookeville, TN 38505 USA

**Keywords:** Faculty online learning community, Research-based instructional strategy, Faculty change, STEM education, Professional development

## Abstract

**Background:**

Adoption and use of effective, research-based instructional strategies (RBISs) for STEM education is less widespread than hoped. To promote further use of RBISs, the propagation paradigm suggests that developers work with potential adopters during the development process, and provide ongoing support after adoption. This article investigates the impact of a faculty online learning community (FOLC) as a professional development mechanism for supporting faculty adopting a research-based curriculum. A FOLC uses video conference technology and online platforms to connect geographically dispersed faculty with similar backgrounds (e.g., physics faculty) and supports their teaching development. In the context of a specific FOLC, this article seeks to determine the outcomes the FOLC achieves, and how.

**Results:**

Analysis of a FOLC meeting identified opportunities for rich, complex social interaction centered on the research-based curriculum. By functioning as a sounding board for ideas, a space to share experiences, a source of affective support, and a venue for troubleshooting, the FOLC mediates the achievement of a range of outcomes related to implementation of the curriculum. Survey results indicate that members feel a sense of community in the FOLC and that it provides encouragement through teaching challenges. Further results indicate participants’ increased confidence in using the curriculum; familiarity with the curriculum structure and content; increased knowledge of pedagogical techniques; reflection on teaching practices in the curriculum; and use of pedagogical techniques aligned with the curriculum’s core principles. Emerging evidence supports more distal outcomes, including student learning, persistence in using the curriculum, reflection in teaching practice across courses taught, and use of research-based pedagogy in other courses.

**Conclusions:**

The propagation paradigm emphasizes the need for ongoing support for adopters of RBISs. The FOLC model provides participating faculty with ongoing support through participation in a community and is an effective support mechanism for adopters of a research-based curriculum. In this study, FOLC members are increasing their knowledge and use of pedagogical techniques in the curriculum-specific course and beyond. This is facilitated by the opportunities in the FOLC for troubleshooting, idea sharing, and receiving encouragement through challenges. This model has the potential to support adopters of additional educational innovations.

**Supplementary Information:**

The online version contains supplementary material available at 10.1186/s40594-020-00268-7.

## Introduction

STEM discipline-based education researchers have developed many effective, research-based instructional strategies (RBISs) that have been shown to lead to improved student learning outcomes^1^ (Freeman et al., 2014; Von Korff et al., 2016). However, the adoption and use of these approaches is far from universal (Laursen et al., 2019; Stains et al., 2018). It follows that focused attention is needed on how instructors learn about, take up, and implement these approaches in order to achieve the improved student outcomes called for in STEM higher education (Olson & Riordan, 2012). More broadly, there is a need for more and better models for supporting faculty’s pedagogical growth and development. This need is acute in situations where faculty are attempting to use approaches that are substantially different from how they themselves were taught. There is a further need for models of support for contingent faculty (lecturers or adjuncts) and isolated faculty (in small departments or lacking local colleagues with shared interests), both of whom may lack access to relevant professional development. Additionally, the COVID-19 pandemic, and the sudden, widespread transition to online instruction, has created an even stronger need for faculty support, and in particular, support in a form that is compatible with remote working and teaching^2^.

Commonly, developers of RBISs use student impact data from pilot implementations to persuade potential adopters via publications, seminars, and workshops. However, an analysis of scholarly work on promoting change in instructional practices in STEM courses noted that there is little evidence supporting the effectiveness of this “develop and disseminate” model as a long-term change strategy for college faculty (Henderson, Beach, & Finkelstein, 2011; Henderson, Finkelstein, & Beach, 2010). While such efforts do increase awareness of innovations, lack of adoption was linked to issues of faculty ownership of their instruction and restrictive situational factors (Dancy & Henderson, 2010; Henderson & Dancy, 2008; Lund & Stains, 2015; Shadle, Marker, & Earl, 2017). Lack of time is also a commonly cited barrier to adoption of RBISs (Brownell & Tanner, 2012; Dancy & Henderson, 2010; Hu, Kussmaul, Knaeble, Mayfield, & Yadav, 2016; Lund & Stains, 2015; Shadle et al., 2017).

Even in cases where faculty do adopt research-based instructional practices, they often modify them to suit their own needs and perceptions. Researchers have discussed the grain size of these modifications and their impact on the effectiveness of an RBIS (Scanlon, Zamarripa Roman, Ibadlit, & Chini, 2019; Stains & Vickrey, 2017). Rather than focus on how similar an adopter’s implementation of an innovation is to that of the original designer’s, it is more appropriate to recognize that faculty will need to make modifications; developers should support adopters so that these changes are consistent with the RBIS (Scanlon et al., 2019). Modification is often an essential step in adoption of an innovation because faculty need to tailor the materials to their context, preferences, and student population (Foote, Neumeyer, Henderson, Dancy, & Beichner, 2014; Hutchinson & Huberman, 1994; Scanlon et al., 2019; Scherr & Elby, 2007). Also, research has found that the more successful change strategies involve interactions with experts in long-term interventions that allowed faculty to reflect on their own teaching (Henderson et al., 2010; Henderson et al., 2011; Holland, Sherman, & Harris, 2018). These studies call for change strategies that account for the complexity of real classrooms (Dancy & Henderson, 2010); are built by collaborative teams of researchers and practitioners; and involve developing reflective teachers and generating buy-in as well as dissemination (Henderson et al., 2010).

A research-based curriculum is one type of RBIS. By curriculum, we refer to materials that can form the basis for a course and that have a coherent pedagogical philosophy and approach throughout, in contrast to standalone pedagogical strategies such as “think-pair-share,” for example. Because a research-based curriculum can require substantial shifts away from status-quo pedagogies, adopters are likely to need considerable support.

In this research article, we investigate the impact of a faculty online learning community (FOLC) as a professional development mechanism for supporting faculty adopting a research-based curriculum. The context for this study is a FOLC for adopters of a specific curriculum, Next Generation Physical Science and Everyday Thinking (Next Gen PET) (Goldberg et al., 2020; Goldberg et al., 2018). First, we provide background on the propagation paradigm, a change strategy employed in developing the FOLC to support Next Gen PET adopters. Next, we present the literature supporting faculty communities and describe FOLCs as a means to support adopters of educational innovations. We then describe the pedagogical characteristics and demands of the Next Gen PET curriculum. We link these characteristics to the types of support adopters will need. We synthesize this background into a conjecture map that lays out a path for answering our research questions: how are the processes that mediate implementation support and achievement of Next Gen PET FOLC goals enacted in the FOLC, and to what extent are the desired outcomes achieved? We next present our research methods. Our results include a transcript excerpt from a FOLC meeting and survey data which indicate how and to what extent the FOLC is supporting adopters. We end with a broader discussion of what this work adds to our community’s knowledge of how to support adopters of educational innovations.

## Background

### Propagation paradigm

In response to the shortcomings of the develop and disseminate model of instructional change, researchers have advocated for a focus on propagating innovations (Henderson et al., 2015). In the extreme form of the develop and disseminate model, the developer focuses on creating the innovation; it is then disseminated to adopters as a final product to implement on their own. In contrast, the propagation paradigm directs developers to work with adopters during the development *and* adoption process (Khatri et al., 2016). Change efforts which subscribe to the develop and disseminate model are predominantly concerned with raising awareness of the efficacy of an innovation, whereas change efforts aligned with the propagation paradigm are equally concerned with the usability of the innovation (Froyd et al., 2017). The propagation paradigm was developed based on a large-scale review of the propagation practices commonly used by STEM education developers and those practices that are used by successfully propagated STEM innovations (such as PhET Interactive Simulations (2020) and Process-Oriented Guided Inquiry Learning, POGIL (Farrell, Moog, & Spencer, 1999)) (Henderson et al., 2015; Khatri et al., 2016). Based on this analysis, the propagation paradigm outlines three aspects of effective propagation plans: interactive development of the innovation, interactive dissemination, and ongoing support for adopters (Henderson et al., 2015; Khatri et al., 2016). In this model, development and dissemination are pieces of a propagation plan, but they are intended to be much more interactive than as enacted in traditional develop and disseminate efforts because the fit of an innovation is a large propagation consideration (Froyd et al., 2017). Moreover, support for adopters acknowledges the variety of contexts in which adopters are situated and this support is the key propagation activity for promoting sustained adoption of an educational innovation. Unfortunately, support for adopters—particularly the type of ongoing, people-based support discussed below—is also the least understood propagation activity and work is needed to identify effective support mechanisms (Khatri et al., 2016). This paper addresses that gap in knowledge.

One tenet of the propagation paradigm is that a successful propagation plan must be designed based on an understanding of the changes required for sustained adoption of an innovation (e.g., curriculum or teaching method) (Henderson et al., 2015). In order to articulate the changes to current practice that will be required of adopters, the propagation paradigm provides guiding questions (Henderson et al., 2015). Developers first need to determine what kind of innovation they have: does it require instructors to make changes in course content, changes in their pedagogy/approach to teaching, both, or neither? Developers also need to consider how flexible their innovation is: do they expect adopters to implement the innovation exactly as is, or do they expect the adopter to make changes to the materials or even the principles behind the innovation? Lastly, developers must consider the resources needed to implement their innovation in terms of collaboration with other instructors or the department, equipment needs, classroom facilities, and personnel required. We address these questions for the Next Gen PET curriculum in the section “Next Gen PET and the changes it requires of adopters.”

Understanding the educational innovation and the change it requires informs the developer of the innovation’s affordances and barriers to adoption. The more changes and resources required by the educational innovation, the more support adopters will need (Henderson et al., 2015). Support for adopters comes in two forms: materials-based support (e.g., users’ guides; website with product materials that can be easily modified by adopters) and people-based support (e.g., individual consultation with adopters; workshops, faculty learning communities). While Next Gen PET has robust materials-based support (e.g., instructor resource site), in this paper we focus on people-based support in the form of a faculty online learning community.

### Faculty communities: a mechanism to support adopters of teaching innovations

Faculty communities have been shown to be effective mechanisms for change. For example, Modeling Instruction, an approach developed in the early 1980s at Arizona State University by Malcom Wells and David Hestenes (Brewe, 2008; Wells, Hestenes, & Swackhamer, 1995), has been widely adopted by high school and university physics instructors. A study of Modeling’s successful dissemination identified building community as a key element (Dancy, Brewe, & Henderson, 2007). Additionally, a study of computer science faculty who were trained in Process-Oriented Guided Inquiry Learning (POGIL) found that in addition to workshops on specific aspects of implementing POGIL, faculty wanted post-workshop support in the form of mentoring and classroom observation from fellow POGIL practitioners (Hu et al., 2016).

Research on effective K-12 teacher development also suggests that key components of change efforts include collaborative environments that develop communities of practice, and sustained professional development (more than 14 h and ideally 30–100 h spread over time) (Darling-Hammond & Richardson, 2009; Lynch, Hill, Gonzalez, & Pollard, 2019). Similarly, in higher education, faculty professional development that involves longer-term participation in a community shows promise (Borda et al., 2020; De Leone, Price, Sabella, & Van Duzor, 2019; Gehrke & Kezar, 2016, 2019; Hayward & Laursen, 2018; Kezar, Gehrke, & Bernstein-Sierra, 2018; Laursen et al., 2019; Pelletreau et al., 2018; Tinnell, Ralston, Tretter, & Mills, 2019). In a community of practice (CoP), people with a common interest come together to fulfill both individual and group goals in a spirit of learning, knowledge generation and sharing, and collaboration (Wenger, McDermott, & Snyder, 2002). Participation takes place at different levels, including legitimate peripheral participation, and learning is framed as moving towards core participation (Lave & Wenger, 1991). Studies of large, sustained STEM reform communities (including the POGIL Project and the BioQUEST Curriculum Consortium) have characterized them as communities of transformation, a particular type of CoP with a guiding philosophy around transforming teaching (Gehrke & Kezar, 2016). Communities of transformation hold particular promise for scaling-up STEM education reform and are more effective than change efforts targeting individual faculty (Gehrke & Kezar, 2016; Kezar, 2011). Outcomes from four successfully sustained communities of transformation focused on STEM education reform include developing individual teaching practice, gaining leadership skills, and networking (Gehrke & Kezar, 2016).

Faculty learning communities (FLCs) can be considered a type of community of practice (Cox, 2004). FLCs support professional development and course transformation and typically consist of 6–15 faculty members. In an FLC, faculty members engage in an active, collaborative program with frequent meetings and activities, often with a curriculum focusing on enhancing teaching and learning. Such FLCs provide professional development in the areas of the scholarship of teaching and learning and community building. Evidence shows that such FLCs increase faculty interest in teaching and learning and provide support to change longstanding instructional practices (Beach & Cox, 2009; Cox, 2004, 2016; Emerson & Mosteller, 2000; Furco & Moely, 2012; Sirum & Madigan, 2010; Thompson, Marbach-Ad, Egan, & Smith, 2016).

CoPs and FLCs can be implemented in hybrid or online forms, with challenges and benefits distinct from face-to-face formats (Barab, MaKinster, & Scheckler, 2003; Brooks, 2010; Gunawardena et al., 2009; Nelson, McKenna, Chavela Guerra, & Pimmel, 2016; Pimmel, McKenna, Fortenberry, Yoder, & Chavela Guerra, 2013; Sherer, Shea, & Kristensen, 2003; Trust & Horrocks, 2017). Faculty online learning communities (FOLCs) translate the FLC model to an online form. Using video conference technology and online chat platforms, FOLCs connect geographically dispersed faculty with similar backgrounds (e.g., physics faculty) to support their teaching development. Given the online nature of FOLCs, they can bring together isolated faculty who may be the only person with a given interest at their institution. This model has been shown to be effective at supporting physics and astronomy faculty in implementing RBISs, enhancing their pedagogical knowledge, and increasing their teaching reflection (Corrales, Goldberg, Price, & Turpen, 2020; Dancy, Lau, Rundquist, & Henderson, 2019).

### Next Gen PET and the changes it requires of adopters

The innovation that provides the context for the FOLC analyzed in this paper is the Next Gen PET curriculum (Goldberg et al., 2018). Next Gen PET is a guided-inquiry, physical science curriculum intended for prospective elementary teachers. In guided-inquiry instruction, students use data (that is provided or that they collect) to explore phenomena and develop scientific concepts; structured activities and the instructor guide them through this process (Moog & Spencer, 2008). The Next Gen PET curriculum covers topics such as forces, energy, waves, physical and chemical changes, and developing models of magnetism and static electricity. The Next Gen PET materials support an experimentally driven, student-centered pedagogy, and are aligned with the Next Generation Science Standards (NGSS Lead States, 2013). The learning goals of the curriculum are achieved by adherence to five core pedagogical principles (each of which is drawn from research on learning) (Goldberg et al., 2020; Goldberg, Otero, & Robinson, 2010; Goldberg, Price, Robinson, Boyd-Harlow, & McKean, 2012):

- Learning builds on prior knowledge
- Learning is a complex process that requires scaffolding
- Learning is facilitated through interaction with tools
- Learning is facilitated through interactions with others
- Learning is facilitated through establishment of certain specific behavioral practices and expectations

Evaluation of student outcomes indicates increases in conceptual understanding with this curriculum (Engelhardt, Robinson, Price, Smith, & Goldberg, 2018; Smith & Wingard, 2019). For more detail on the curriculum, see the instructor website (Goldberg et al., 2020).

To determine the change from current practice the curriculum requires adopters to make, we consider the guiding questions provided by the propagation paradigm (described above and in (Henderson et al., 2015)). Namely, what kind of innovation is Next Gen PET; how flexible is it; and what resources are needed to implement the curriculum?

The Next Gen PET curriculum incorporates non-traditional course content and pedagogy. The content included in the curriculum deviates from widely used physical science textbooks (e.g., Hewitt, 2015) including units on models of magnetism and static electricity, presenting energy before forces, and including engineering design and activities on teaching and learning. There is focus on deep conceptual understanding of a few big ideas rather than many detailed topics and typical end-of-chapter problems. Pedagogically, the curriculum makes extensive use of active learning techniques. It is expected that students will work in groups to draw on evidence gathered in class and (with guidance) collaboratively come up with the main ideas. The instructor takes on the role of facilitator, as opposed to content provider. This requires a significant shift in an adopter’s approach to teaching if they are used to instructor-centered, lecture-based practice. Even if an instructor is familiar with active learning pedagogy, we expect that implementing an active-learning, student-centered curriculum like Next Gen PET provides a rich opportunity for pedagogical growth, as has been shown by others (Holland et al., 2018; Horn & Kane, 2015).

The curriculum is semi-flexible: individual activities are not available in editable format, but instructors have flexibility with respect to content and implementation format. For example, the materials are modular and available at the unit level allowing the instructor to choose which topics to cover. Additionally, distinct versions of the curriculum are available for studio classrooms and lecture hall settings. There is an expectation that adopters will adhere to the large-scale activity structure and design provided by the curriculum. However, it is expected that instructors will differ in the fine-grain sized implementation details of these elements in order to fit their context and experience. For example, engaging students in reaching consensus through a whole class discussion is one of the activity structures built into the curriculum, but instructors can enact these whole class discussions in a variety of ways: framing them around a clicker vote, using a round-robin whole group share out, or other facilitation methods.

Depending on the current teaching situation of adopters, Next Gen PET requires minimal to considerable resources, which in the propagation model includes material resources, personnel, and buy-in from colleagues and students. If an adopter is changing from a lecture-style course to a studio version of Next Gen PET, they will need different classroom facilities, equipment, and buy-in from the department. On the other hand, if they are adopting the Next Gen PET version for lecture hall settings, they may only require additional personnel, such as a learning assistant (Otero, Pollock, McCray, & Finkelstein, 2006). Either way, given that the curriculum is a departure from the style of teaching and learning instructors and students are used to, adoption will require buy-in from students.

Based on this analysis, we expect that adopters of Next Gen PET will need considerable pedagogical skill at facilitating guided-inquiry learning. Together with the partial flexibility of the curriculum and the resources required to adopt Next Gen PET, the propagation paradigm would predict that Next Gen PET adopters will benefit from a substantial amount of ongoing implementation support. The characteristics of the curriculum indicate the specific types of support adopters may need; we detail this support in Table 1. Recognizing the challenge of this implementation work, faculty will also need affective support (i.e., encouragement and moral support), during this process. Affective concerns (lack of confidence, fear, lack of motivation) have been identified as barriers to adoption of RBISs (Sturtevant & Wheeler, 2019, and references therein), and positive affective impacts have been identified as an important outcome of participation in a FOLC (Corrales et al., 2020; Dancy et al., 2019).

**Table 1:** Types of support Next Gen PET adopters may need

Characteristic of the curriculum	Type of implementation support^a^
Non-traditional course content	→ Anticipating student questions and appropriate responses based on reordering of topics → Adjusting expectations for content coverage and learning objectives → Recognizing that students’ development of ideas will occur over a longer time than is traditional
Non-traditional pedagogy	→ Troubleshooting difficulties which arise from student group work → Examples of different facilitation strategies → Ideas on creating classroom norms that support pedagogy
Semi-flexible: Modular design, but with pedagogical coherence	→ Assistance choosing modules and units to cover, pacing guidance → Understanding connections and sequencing among topics, and implications of choices about what to include
Semi-flexible: choice in implementation format	→ Reflecting on students’ needs and available resources to select studio or lecture format for local situation; creative combining of formats
Resources: equipment, facilities	→ Identifying available resources → Logistical support
Resources: collaboration, staffing, buy-in	→ Reflecting on programmatic needs → Justifying class practices and policies to students and colleagues

Based on an analysis of the curriculum’s characteristics guided by the propagation paradigm (Henderson et al., 2015)

^a^The types of support are examples, not an exhaustive list

Some of these needs can be met by the extensive materials-based support provided by the Next Gen PET instructor resource site (Goldberg et al., 2020). Materials include pacing guidance, sample course sequences, copies of student materials, instructor presentation slides (for lecture-based formats), test banks, equipment lists, ordering information, videos of classroom interactions, and more. These materials help provide some of the implementation support needed by adopters of the curriculum. However, the Next Gen PET developers did not expect these materials to provide sufficient support for adopters; the team additionally provides people-based support in the form of a FOLC for adopters. The Next Gen PET FOLC was designed to provide the implementation support listed in Table 1 via opportunities to troubleshoot teaching challenges, share information and resources, have a sounding board for ideas, explore pedagogical concepts in the context of problems of classroom practice, and have a source of affective support (e.g., encouragement and moral support). We refer to these as “mediating processes” (this term is discussed in more detail in the next section). The FOLC developers expect that, through engagement in these mediating processes (that is, through participation in the FOLC), faculty will:

- Become more familiar with Next Gen PET structure, content, and materials
- Develop greater knowledge of pedagogical techniques
- Gain confidence in using curriculum
- Reflect on their Next Gen PET teaching practice
- Expand their use of pedagogical techniques aligned with Next Gen PET core principles

To guide this research study, we developed a conjecture map, presented in the following section, that formalizes and makes explicit the FOLC developers’ approach. Based on this conjecture map, the study proceeded in two steps: first, verify that the mediating processes are occurring. Second, test the hypothesis built into this model. We present specific research questions after describing the conjecture map.

### Conjectures and research questions

**Fig. 1:**
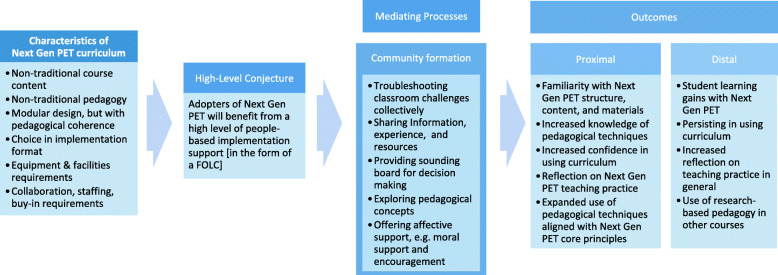
Conjecture map. Given the characteristics of Next Gen PET, adopters of the curriculum will benefit from extensive, ongoing people-based implementation support. This support is provided by the five mediating processes. The theoretical conjecture, represented by the third arrow in the figure, proposes that if the mediating processes are provided by the FOLC, we expect to see participants reaching the outcomes shown (the goals for FOLC participants). Achievement of these outcomes indicates the FOLC is helping meet the needs of adopters

To illustrate the process that underlies the design and implementation of the Next Gen PET FOLC, we constructed the conjecture map shown in Fig. 1,^3^. This conjecture map illustrates how the mediating processes are intended to link starting conditions and outcomes in the Next Gen PET FOLC, and we use it as a tool for directing our analysis in order to answer our research questions. Given the characteristics of the curriculum and associated forms of implementation support needed (see Table 1), the high-level conjecture underlying the FOLC is that adopters of the curriculum will benefit from extensive, ongoing people-based implementation support. The FOLC was designed to provide this support by engaging faculty in the mediating processes listed above and in Fig. 1. In the conjecture map, these mediating processes are associated with participating in a professional community. Moreover, participation in such a community provides an opportunity for longer-term, ongoing support while faculty are engaging in implementation—in contrast to (for instance) short-term workshops that occur before teaching. The theoretical conjecture embedded in the map is that, if the FOLC provides opportunities for these processes to occur, participants should reach the outcomes listed in Fig. 1. These outcomes represent the FOLC’s goals for participants. Further, achievement of these outcomes will indicate that the FOLC is helping meet the needs of adopters. For example, if an adopter troubleshoots their teaching challenges with a group of fellow adopters, is able to share and receive resources for teaching the curriculum, can talk through teaching decisions with the group, and receives encouragement along the way, one would expect to see that they have increased knowledge of pedagogical techniques, and in the long term they may implement the techniques in their non-Next Gen PET courses. The FOLC, then, would be meeting the need of adopters to enact the pedagogy required by Next Gen PET.

Of course, some of the mediating processes could be observed in adopters’ use of material-based support. The propagation model does not suggest that people-based support is “better” than material-based support; in fact, the two types of support should complement each other. However, in assessing the people-based support the Next Gen PET FOLC provided, an absence in observing one of the mediating processes would indicate a gap in the designed support.

Similarly, one would expect to see some of the listed outcomes from curriculum adopters who did not participate in any sort of people-based implementation support. In the following analysis, we will not argue that the outcomes we observe are solely due to participation in people-based implementation support. Indeed, we cannot disentangle the coincident experiences of teaching the curriculum while participating in the FOLC. Instead, we will argue that such support, provided by the set of mediating processes (indicated above), has contributed to the achievement of any outcomes that match those listed in Fig. 1. That is, observing the mediating processes *and* outcomes provides a plausible argument for the connections illustrated in the conjecture map.

The research questions addressed in this paper are:

- RQ1—Occurrence of mediating processes: How do the mediating processes listed in the conjecture map (Fig. 1) get enacted in the Next Gen PET FOLC?
- RQ 2—Testing the theoretical conjecture: If the FOLC provides opportunities for these mediating processes to occur, to what extent are the outcomes listed in the conjecture map (Fig. 1) achieved? More specifically, 2 years into the lifetime of the FOLC, to what extent are the proximal outcomes achieved, namely, familiarity with Next Gen PET structure, content, and materials; increased knowledge of pedagogical techniques; increased confidence in using curriculum; reflection on Next Gen PET teaching practice; and expanded use of pedagogical techniques aligned with Next Gen PET core principles?

In answering these questions, we aim to address the call of the propagation paradigm to identify productive support mechanisms for adopters of teaching innovations. We focus specifically on the support the FOLC provides Next Gen PET adopters because people-based support has potentially far reaching impact for helping faculty learn, become more reflective, and increase their pedagogical sophistication. Additionally, unlike material-based support, people-based support can provide adopters encouragement and motivation through their implementation. From a research perspective, understanding the processes of support is a very rich area of inquiry. While these questions are focused on the Next Gen PET FOLC, answering them helps speak to the general utility of the FOLC model for supporting adoption of a range of teaching strategies and materials.

## Methods

### Study context

The Next Gen PET faculty online learning community (FOLC) is an online faculty learning community for faculty who teach physics or physical science courses for future elementary teachers, general education students, or a mixed audience, using the Next Gen PET materials. Many of these faculty are the only person at their institution teaching physics or physical science for prospective elementary teachers, and the online nature of the FOLC provides an instructional community despite their geographic isolation. The FOLC is designed for faculty to learn from each other, to share resources and collaborate to improve their instruction, to study student thinking, and to conduct classroom-based research. Faculty meet regularly by videoconference in small groups to discuss practical issues, facilitation strategies, and student learning. In these meetings, participants have the opportunity to discuss problems of practice and gather feedback from the group. Because FOLC members are teaching the Next Gen PET curriculum concurrently with their participation in the FOLC, they can bring pressing concerns to the group and receive timely solutions. These discussions may include encouragement to stick with the teaching changes one has made and ideas on concrete techniques to try to solve the implementation challenge at hand. In addition to the videoconferences, online communication and file sharing tools (Slack, Google Docs) support collaboration between meetings. These platforms offer an easy way to share curricular resources and also act as a venue for soliciting timely feedback. Both the synchronous videoconferences and the asynchronous communication facilitate the mediating processes described in Fig. 1.

The Next Gen PET FOLC consists of 50 participating faculty members organized into five clusters. Each cluster consists of 6–10 novice Next Gen PET instructors^4^ and 2 experienced faculty who serve as leaders. These lead faculty (“cluster leaders”) are experienced with the curriculum and pedagogy and they facilitate their cluster’s meetings and interactions. The Next Gen PET FOLC is intended to be an ongoing, multi-year community (in contrast to a cohort model, where faculty participate for a semester or year). The community started in spring 2017 with the ten cluster leaders meeting regularly with the Next Gen PET FOLC project team. With the guidance of the project team, the cluster leaders developed expertise with Next Gen PET as they implemented it in their courses and received preparation and planning guidance to lead their clusters the following term.

In fall 2017, the community added the larger group of members (40 novice Next Gen PET instructors, split into 5 clusters and led by the cluster leaders). The project is funded to continue through the 2020–2121 academic year. In summer 2017, 2-day, in person workshops introduced participants to the Next Gen PET curriculum and to each other. During 2017–2018, the FOLC focused on faculty’s successful implementation of their courses and has since emphasized collaborative projects (e.g., conducting classroom research) and exploring deeper problems of practice. For the videoconference meetings, cluster leaders were given overall goals as well as suggestions for meeting structure (e.g., having members share a “high” and a “low” from their teaching that week; getting updates from members quickly using a round-robin format), but the cluster leaders were encouraged to respond to the group’s needs and interests. That is, the topics of a meeting were not strictly prescribed by the project team and the different clusters did not have identical meeting agendas. Generally, members did not have any readings or “homework” to complete in preparation for a meeting.

### FOLC participation rates and topics of discussion

To convey the level of engagement and focus in the community, we briefly describe participation rates and the topics discussed. Logs of online interactions and video recordings (or transcripts) of online video conference meetings were reviewed to track participation and identify topics of discussion. Clusters meet approximately every 2 weeks, and during the 2017–2018 academic year the total number of meetings per cluster varied from 13 to 17. Over half of the FOLC members participated in 9 or more meetings, and on average each cluster had more than half of its members attend each meeting. For the purposes of formative feedback to support community facilitation, a member of the project team reviewed cluster meeting transcripts and Slack logs for two of the clusters from 2017 to 2018. These clusters were selected to represent each of the two implementation formats (one cluster was implementing Next Gen PET in lecture-style classrooms and the second cluster was teaching in studio-style classrooms). The goal of this review was to describe (in broad terms) the topics of the FOLC cluster discussions. Through this review, the team member identified four emergent categories of discussion: course management or logistics, pedagogy, operation of the FOLC itself, and miscellaneous topics. Discussions of which units members are teaching, coverage and scheduling, test administration, and equipment issues were counted as management/logistics. Discussions related to issues such as student thinking, student engagement and affect, facilitation (managing groups and class discussions), adding new content, formative assessment, and design of exam questions were counted as pedagogical. For example, the transcript excerpt in the section “Evidence from a FOLC meeting” under Results Part 1 is considered a pedagogical discussion. Operation of the FOLC itself included discussions such as adding new group members. The Miscellaneous category captured greetings, conversations about general professional concerns (e.g., tenure) or upcoming professional conferences, and discussion of non-Next Gen PET courses. Together, management/logistics and pedagogy were by far the most common topics of discussion, comprising about 95% of the topics. These two categories were discussed with similar frequency, although there was some variation by cluster and from the first to the second year of the FOLC. As indicated above, this review was conducted not as a formal research effort but rather to provide the project team and cluster leaders with feedback in the form of large-grain size categories describing the discussion topics of the clusters. We present it here merely to provide a general sense of the topics of FOLC discussions.

### Data sources and analysis

This study was conducted using both qualitative and quantitative methods. Research question 1 was addressed through a concurrent mixed design (Teddlie & Tashakkori, 2006) involving two strands of research design (investigating the types of processes that can occur during a meeting and investigating participants’ sense of community). The first strand used qualitative methods (analysis of a meeting transcript) and the second strand used quantitative methods (analysis of surveys responses related to sense of community). These two strands were pursued at the same time (concurrently) and minimally informed each other (e.g., the survey questions were not designed based on analysis of the transcript). Inferences from these two strands were synthesized to draw overall inferences related to research question 1. Research question 2 was addressed with a quasi-mixed, single research strand (investigating outcomes of participation in the FOLC) design, using quantitative analysis of survey results and qualitative analysis of open-ended survey responses.

Data sources collected through the overall Next Gen PET FOLC research efforts included video recordings and transcripts of FOLC meetings, logs of participation in online communication platforms, interviews with select faculty participants, and surveys of participants. For the present study, we draw on survey data and the transcript of one FOLC meeting. Analyses involving the other data sources have been reported elsewhere, or are in preparation (Corrales et al., 2020; Corrales, Goldberg, Turpen, & Price, 2018; Lau, Corrales, Goldberg, & Turpen, 2019; Turpen, Goldberg, Corrales, & Price, 2018).

### Surveys

Surveys were conducted before and after an initial, in-person workshop (summer 2017), and at the end of the second year of the FOLC (spring 2019). Surveys included a mix of closed and open-ended questions, and all surveys were administered by Horizon Research, Inc. (HRI), the external evaluator for the project. The workshop surveys addressed participants’ perceptions of the workshops, their concerns and readiness regarding teaching Next Gen PET, their preparation to participate in the FOLC, and their views about teaching. Cluster leaders received this survey as well because they are also expected to learn and grow professionally from their participation. Response rates for the pre- and post-workshop surveys were approximately 95%.

The spring 2019 survey was designed by the research team in collaboration with HRI. The survey was designed both to serve research purposes and to provide formative feedback to the FOLC project leadership. The development of the survey preceded the construction of the conjecture map and the present research study. The full survey is included in the Supplemental Material. Again, cluster leaders received this survey along with the other participants; the response rate was approximately 80% (39 out of 48). The results presented in this paper come from both the cluster leaders and the novice Next Gen Pet instructors (i.e., the results are not disaggregated by participation role in the FOLC. For our analysis, cluster leaders and novice Next Gen PET instructors are treated as a single group). In this survey, the first item asked participants to rate to what extent each of 15 potential benefits had occurred for them as a result of participating in the FOLC during the 2018–2019 academic year. The ratings were on a four-point scale: not at all, minimally, moderately, to a great extent. Participants provided a rating for each of the 15 statements. The 15 specific potential benefits were based on (1) impacts identified through interviews with participants of a FOLC serving new physics and astronomy faculty (Dancy et al., 2019), (2) the research team’s ongoing analyses of Next Gen PET FOLC meetings, (3) interviews conducted in summer 2018 with a selection of Next Gen PET FOLC members after their first year in the FOLC, and (4) the goals for FOLC participants. For example, some of the benefit statements were designed to test impacts (like time and efficiency) in the Next Gen PET FOLC environment that had been identified through research on a different FOLC (Dancy et al., 2019). Additionally, we hoped one of the impacts of participating in the FOLC would be increased confidence and motivation in teaching, and so three of the survey items had to do with attitudes toward teaching. Likewise, we expected the FOLC to impact participants’ teaching practices, and some of the benefit statements were intended to gauge this impact.

For the purposes of analysis, we performed a post-hoc grouping of the statements into thematic categories: Teaching practice & pedagogy; Attitude toward teaching; Student impact; Time & efficiency; and Community. Participants did not see the statements grouped into these categories when they filled out the survey; the categorizing was part of our analysis process. First, one of the authors (A.L.) labeled each of the 15 statements with a short descriptor summarizing what it was about. For example, the benefit statement “I have incorporated ideas from the FOLC into my teaching” was labeled as “implementation change” and the statement, “I have been introduced to new concepts (about teaching and learning) that are helpful for thinking about my ongoing teaching work” was labeled as “understanding pedagogical concepts.” Across the 15 benefit statements, 10 summary labels were identified. Following this first step, two of the authors (E.P. and A.L.) discussed the 10 labels and collapsed them into the five thematic categories listed above. These categories were semi-emergent and selected to describe the different types of benefits (impacts) succinctly, while preserving distinctions among the nature of impacts (e.g., “Community” describes relationships with others while “Teaching practice and pedagogy” pertains to teaching skill and ways of thinking about teaching). When sorting the items into the categories during the analysis phase, the two authors discussed each item until they reached complete agreement on its categorization. The remaining authors reviewed and agreed with this categorization.

The spring 2019 survey also asked a number of open-ended questions that were intended to allow participants to elaborate on their responses to the close-ended (rating) questions and to check their interpretations of the close-ended items. The following open-ended items pertain to the current study: (1) Please briefly describe the most significant impact(s) of participating in the FOLC; (2) What did you find most valuable about your involvement in the FOLC? The impact question received 36 responses (92% of the 39 respondents) and the value question received 33 responses (85% of the respondents). For both questions, we reviewed responses, coding for the main topic(s) of the response. Coding categories included (a priori) the five thematic categories identified in the 15 benefit statements (described above); we also allowed for categories to emerge (e.g., some participants talked about the impact of the curriculum itself). We specifically analyzed the statements for evidence of the five thematic categories described above because we wanted to identify illustrative examples of these benefits. In the results sections of this paper, we will present responses to these open-ended questions as a means to explain the close-ended survey results. We do not report detailed information on the frequencies of different codes in the open-ended responses because the intent of the questions was mainly to seek out explanation, rather than prevalence.

Thus, most of the data presented in this paper is from participants’ self-reporting of their FOLC experience (via the surveys). This self-report data is particularly useful for learning about aspects of participants’ experiences for which there is no direct measure or where we are most interested in their personal insights. For example, in this study, a participant’s perceptions of their teaching mindset or sense of community were most important to us. Perceptions influence practice and are thus important to collect. Note, because some of the project researchers are also involved in the implementation of the FOLC, we recognized that participants may not feel comfortable being completely candid in their feedback. This is why the project arranged for an external evaluator (HRI) to collect these data. Project participants were assured that the evaluator would keep their data anonymous, making it less likely that participants would withhold their honest feedback in order to please the project leaders.

### Transcript excerpt from FOLC meeting

To address research question 1, we wanted to document examples of mediating processes occurring during a FOLC meeting. At this stage of the research, our goal was not to make claims about the prevalence of such processes, but rather to determine what is possible within this context. Thus, we selected for analysis a meeting excerpt that had previously been identified by other members of the research team (for unrelated purposes) as an exemplar of a pedagogically focused discussion. Authors A.L. and E.P. then independently coded the transcript for the five mediating processes listed in the conjecture map (troubleshooting teaching challenges, sharing information and resources, providing a sounding board for ideas, exploring pedagogical concepts in the context of problems of classroom practice, and offering a source of affective support). We then compared our coding and found only one discrepancy; A.L. coded one portion of the transcript at only one of the mediating processes, while E.P. found that an additional mediating process applied. This discrepancy was due to an inadvertent omission by A.L., who upon re-reading the segment readily agreed that both meditating processes were represented in the segment. This coding was then reviewed and agreed to by the remaining authors.

### Participant demographics

Participants in the Next Gen PET FOLC were recruited at conferences, through emails to department chairs, direct emails to adopters of the Next Gen PET curriculum (or its earlier versions), and by word of mouth. This section describes the characteristics of the faculty participating in the Next Gen PET FOLC. This information was provided by faculty via surveys (e.g., a demographics survey administered in December 2019) or, in the case of institutional type, obtained from public sources.

Information on the institutional context of participants is provided in Table 2. Participants are located at masters granting (60%), doctoral (27%), primarily undergraduate (5%), and 2-year college (7%) institutions. Most participating faculty are located at public institutions (76%). In the December 2019 demographics survey, most of the *N* = 42 respondents (response rate of 85%) reported that they spend a majority of their time on teaching responsibilities (mean of 74% of work time on teaching), that their institution gives teaching somewhat (29%) or much (45%) higher priority than research, and that their own teaching work is valued by their department (24% moderately, 71% greatly). Respondents described departmental affiliations with physics or physics and astronomy (*n* = 28/42, 67%), natural or physical sciences (12/42, 29%), or education/science education (12/42, 29%). Ten faculty (24%) listed affiliations with departments in both the sciences and in education.

**Table 2:** Institutional context of faculty participating in the Next Gen PET FOLC

Institution type (*n* = 55)	
Masters granting	60%
Doctoral granting	27%
Two-year college	7%
Primarily undergraduate	5%
Public	76%
Departmental affiliation (*n* = 42)	
Physics/Physics & Astronomy	67%
Natural or Physical Science	29%
Education/Science Education	29%
Both a science department and education	24%

Institution type information was obtained from public sources for the 55 total participants. (The community has a total of 50 current members, but 5 of the original members had to drop out and were replaced by 5 new members.) Departmental affiliation was collected via a demographics survey administered to participants in December 2019. There were 42 responses to the survey (85% of participants)

Two-thirds of responding faculty described their position as tenured or tenure track. Non-tenure track participants are mostly in full-time positions (81%), with contract lengths varying from 1-year or less (50%), 2–3 years (13%), 5 years (19%), or > 5 years (19%). It is noteworthy that the FOLC is engaging non-tenure track faculty, as they teach a significant portion of university courses and often have limited professional development opportunities (Curtis, 2019).

In order to describe who is participating in the community, who the project is reaching, and to enable comparison with other studies, participating faculty were asked to describe how they identify with various demographic categories (Table 3). The *n* = 42 respondents correspond to 85% of the participating faculty, thus representing a large majority of the community—but not its entirety. Some of these categories, such as gender, are frequently reported (for instance, by the American Institute of Physics Statistical Research Center). Other categories, such as sexual orientation and race, are not typically reported, but are included here in response to calls to do so (Ackerman et al., 2018; Kanim & Cid, 2020; Parks & Schmeichel, 2012). By reporting these demographics, we make visible some of the characteristics of physical science faculty that are often kept invisible. In addition to recognizing the variety of ways STEM faculty self-identify, this information will enable more accurate comparison of results across studies of STEM faculty communities, which may have different demographic compositions. Identity can affect one’s experience in a community^5^, so this demographic information is important context to report.

**Table 3:** Demographics of faculty participants

Race and ethnicity	
White	86%
Asian/Asian American	12%
Black, African, or African American	2%
Hispanic, Latino, or Spanish origin	2%
Gender identity	
Female/Male	50%/50%
Cisgender	7%
Sexual identity	
Heterosexual/straight	98%
Bisexual	2%
Disability/ability status	
Do not identify with disability or impairment	71%
Sensory Impairment (vision or hearing)	14%
Mental health disorder	7%
Learning disability (e.g., ADHD, dyslexia)	5%
Mobility impairment	2%
Temporary impairment due to illness/injury	2%

Information collected through a demographics survey administered to participants in December 2019. There were 42 responses to the survey (85% of participants)

Racially and ethnically, respondents described themselves as white (86%), Asian/Asian American (12%); Black, African, or African American (2%); and Hispanic, Latino or Spanish origin (2%). Half of respondents described their gender identity as female, half as male, and 7% said they identify as cisgender. Regarding sexual identity, 98% described themselves as heterosexual/straight. Most respondents reported they did not identify with a disability or impairment (71%), but 14% reported a sensory impairment (e.g., vision or hearing). In order to ensure the confidentiality of faculty responses to our surveys, the results reported in this paper will not be disaggregated based on demographics^6^.

The ten cluster leaders had a range of experience with the curricula on which Next Gen PET is based (Goldberg et al., 2012; Goldberg, Robinson, Kruse, Thompson, & Otero, 2007; Goldberg, Robinson, & Otero, 2008), but little-to-no experience with Next Gen PET itself. However, the cluster leaders joined the FOLC and began teaching with Next Gen PET at least one semester before the other FOLC members. In their applications to participate in the FOLC, many of the 40 novice Next Gen PET instructors (non-cluster leaders) reported a lack of experience with the curricular model, with 24 not having taught one of the curricula on which Next Gen PET is based. Of those 24, eight reported not having prior experience with other guided-inquiry materials. Thus, of the 40 novice Next Gen PET instructors, 20% had no prior experience with guided-inquiry and 60% had not taught with a previous version of Next Gen PET.

### Results part 1: mediating processes

In order to address our first research question on *how the mediating processes listed in the conjecture map* (Fig. 1) *get enacted in the Next Gen PET FOLC*, we will consider evidence of the mechanisms in the FOLC that facilitate change. In particular, we explore this question by presenting an excerpt from a FOLC meeting that illustrates how the mediating processes can play out in the FOLC and demonstrates the linking between process and outcome. We then provide survey results on participants’ perceptions of the FOLC community and the role it plays in addressing their needs. Recall from Fig. 1 that the Next Gen PET FOLC was designed to provide the following mediating processes: troubleshooting teaching challenges (abbreviated and bolded below as “**troubleshooting**”); sharing information, experiences, and resources (“**sharing experience**”); having a sounding board for ideas (“**sounding board**”); exploring pedagogical concepts in the context of problems of classroom practice (“**exploring pedagogical concept**”); and having a source of affective support, e.g., encouragement and moral support (“**affective support**”).

### Evidence from a FOLC meeting

Below, we include a transcript of 4-min excerpt from a cluster meeting which occurred midway through the first year of the FOLC. Our purpose in presenting this excerpt is to give a view inside a Next Gen PET FOLC meeting and, most importantly, illustrate how the mediating processes can occur in a FOLC meeting. This excerpt is an example of a conversation that contains many examples of mediating processes in a short period of time. Further, it illustrates the types of deeper learning opportunities a FOLC can provide. With this excerpt, we explore what is possible in terms of the enactment of mediating processes, but we do not make claims about the prevalence of these processes.

The meeting the excerpt is extracted from opens with social pleasantries (e.g., “How are you?”) and then moves on to members updating each other on what is going on in their classes. Wallace is the second member to provide an update. His students are working on the Magnetism Unit of the Next Gen PET curriculum, which guides students through the process of building a model to explain magnetic phenomena. Wallace first shares, “A lot of the students are having difficulty predicting what will happen based solely on the model that they have come up with...they’re struggling with the sequence of model prediction, test, revision.” A number of cluster members normalize this issue, saying they have experienced it too, and they suggest some potential solutions (an example of the mediating processes of **affective support**, e.g., moral support and encouragement, and **troubleshooting**, among others). Then Wallace shares a second issue he is encountering. In the following transcript, turns are identified based on a change in speaker and are numbered sequentially in the order in which they occurred in the conversation. No turns of talk are omitted (i.e., there are no gaps in the transcript).

**Table:** 

Turn	Speaker	Transcript
T1	Wallace	The other issue I have, which is ... I think this mainly arises because we meet only twice a week for such a short time, so the module gets drawn out over a few weeks, is some students go away and Google how magnetism works, and so suddenly they’ll be talking about domains. Okay, so this is kind of skewing the process.

By students searching the internet for how magnetism works and bringing those ideas into class (without basing it on any evidence developed during class), Wallace worries that they are undermining the learning process intended by the curriculum (Turn 1, T1). The students’ behavior is a problem for Wallace because it does not align with the pedagogy behind the Next Gen PET curriculum. He believes the students’ actions may be derived from their local, situational factors (class meeting duration and frequency “drawing out” the module).

Wallace’s first-person narrative account of his classroom launches the cluster into a discussion of this implementation challenge. In the exchange that follows, Courtney, a cluster leader, **shares** her **experience** with this issue:

**Table:** 

Turn	Speaker	Transcript
T2	Courtney	Yeah, but I’ve found that in my experience anyway, if they go away and they come back with domains that they usually don’t have any idea how they actually work.
T3	Wallace	No, yeah, that’s true.
T4	Courtney	They try to use domains to explain whatever, and they can't, and so the rest of the class is like, “Well, never mind that. We’ll just forget ... ”
T5	Wallace	I think that is true. They google the answer, but they’re not really quite understanding what’s going on still. I’m not too worried about that. It was just funny when they suddenly start pulling out these words.

Courtney responds to Wallace’s challenge by normalizing the problem (she has faced it too) (T2, T4). This is another example of the **affective support** the FOLC provides, by helping participants realize that other faculty have faced, and overcome, similar difficulties. Her normalizing seems to assuage Wallace’s concern, as he responds, “I think that is true...I’m not too worried about that” (T5). Courtney replays what has happened in her classroom when students introduce information they looked up outside of class (T2, T4). She provides her personal experience that students will stick to the model building process of the curriculum because students do not understand the information they find online. In this way, she reframes the problem as potentially not a problem at all; she offers the perspective that students looking things up might not skew the process that much. Though unstated here, we suspect that Courtney’s assessment relies on the assumption of a classroom culture that places primacy on models developed through experimental evidence. Wallace seems to agree with Courtney’s interpretation.

Carter, another cluster leader, adds a second perspective:

**Table:** 

Turn	Speaker	Transcript
T6	Carter	Kind of seems like it’s evidence about the students’ epistemology, like I feel like they don’t have very sophisticated views about what it means to understand something.
T7	Courtney	Oh, they don’t.
T8	Carter	Because in science context, what it means to understand something, and so for them understanding means like knowing the term or being familiar with the term when we’re trying to give them an experience that’s a much different view of what it means to understand something, and there’s a tension there.
T9	Courtney	Yeah, they want to memorize. "I must memorize."

Carter introduces the pedagogical concept of “epistemology,” and posits that students’ internet searching is due to their notion of what is sufficient to understand a scientific concept (T6). He **explores this pedagogical concept** further, hypothesizing that the students think knowing a word is the same as understanding what it means (T8). He identifies the students’ epistemology as misaligned with the view of understanding underlying the Next Gen PET curriculum and science more generally. Carter goes on to **share** a personal **experience** he has had of this (T10):

**Table:** 

Turn	Speaker	Transcript
T10	Carter	I had a student after the quiz, he was in my office complaining, different student than the other one that I mentioned earlier, he’s going [on] about how he understands everything in this class, because after all this class is like baby physics, and he learned it all in high school, but he just can’t explain it the way that I want him to. He went on and on and on and on. I tried to provide some, “Have you thought about maybe writing an outline of the key bullet points that you wanna hit in your explanation, and only then start ... ” I just tried everything I could think of to get him to reflect on maybe I don’t fully understand it. My struggles are evidence that I don’t fully understand it. Every time I tried to hand it back to him, he just kept handing it back to me. Like, “No, this is so easy, and I just ... yeah, I can’t explain it the way you want.” Oh God, just leave.
T11	Courtney	Right.

Carter’s idea about students’ epistemology being the root cause of their online search for information is at least partially based on his experience with students thinking they understand a physics concept because they know the terminology, even if they cannot explain it.

Another cluster member, Yin, then asks for some clarification on the conversation (T12):

**Table:** 

Turn	Speaker	Transcript
T12	Yin	What is domain? I’m sorry I don’t think I fully understand. What kind of question that they google?
T13	Wallace	Oh, this is they’re googling ... they’re basically trying to google how ferromagnetism and iron gets magnetized, so they come across the idea of magnetic domain, certain small regions that are polarized in the magnet. They generally don’t really understand what that means. They just start using these words because it’s something they’ve seen.

Yin is new to the curriculum and her question provides Wallace the opportunity to synthesize what Courtney and Carter have shared (T13). Yin then receives **affective support** in the form of encouragement from the community to try the magnetism unit in her course (T14–T16):

**Table:** 

Turn	Speaker	Transcript
T14	Carter	Yin, have you taught the magnetism unit?
T15	Yin	No, but I am very much looking forward to it.
T16	Carter	Yeah, it’s so awesome. I would encourage you ... you gotta find a way-
T17	Wallace	Despite these problems, I think it is really good, and I think the students are getting a lot out of it.

While Wallace has encountered some challenges in implementing the magnetism unit, this has not dissuaded his use of the unit because he sees students learn a lot (T17).

This episode offers one example of a FOLC member **troubleshooting** a problem of practice with the group. Wallace is able to describe his concern (T1) and the ensuing conversation provides a **sounding board** for sharing and developing different ideas about the issue. Courtney normalizes his situation by sharing her own experience with the “googling issue” (T2, T4). This provides **affective support** because it offers reassurance that the problem is a common one. Carter **explores the pedagogical concept** of students’ epistemology as the potential source of the issue, and supports the idea with an experience from his class (T6, T8, T10). In surfacing multiple perspectives on the issue Wallace is facing, this conversation affords all participants the opportunity to expand their pedagogical understanding. Participants who are currently facing the implementation challenge (Wallace), participants who may face it in the future (Yin), and participants who have encountered it in the past (Carter and Courtney) can all learn something from the discussion. From Wallace’s contributions to this conversation, we can specifically see *how the mediating processes lead to learning outcomes*; it is in troubleshooting this issue and having a sounding board for new ideas that Wallace is able to engage in reflection (one of the FOLC’s intended outcomes, see Fig. 1) and evolve from his stance in T1 that searching the internet for information is a problem to his revised perspective in T13.

This meeting excerpt provides just one glimpse into how the mediating processes can unfold during FOLC meetings. This conversation was interactive (four of the six meeting attendees contributed), but we know from the project team’s informal monitoring of FOLC meetings that conversations can range in the number of voices heard. As participants share implementation challenges (and successes), members can respond in a variety of ways that either open-up or close-off the conversation to others’ contributions. In many FOLC meetings, we see people offer specific solutions, ask probing questions, share their own experience, normalize the experience of others, offer encouragement, bounce around ideas, and develop pedagogical concepts (Corrales et al., 2020; Lau et al., 2019; Turpen et al., 2018). The frequency and quality of these different interactions can vary, however, and is the subject of ongoing study (Lau et al., 2019). Regardless of the exact topic of conversation and the nature of responses, the above example demonstrates that FOLC meetings can provide the opportunity, through the mediating processes, for rich, complex social interaction centered on the Next Gen PET curriculum.

### Evidence from spring 2019 survey

Responses to the spring 2019 survey offer further evidence of the presence of these mediating processes in the FOLC as well as their role in faculty participants’ learning.

All of the mediating processes listed in the conjecture map (Fig. 1) can be provided by participating in a professional community. In such a community, members typically have opportunities to discuss solutions to problems of practice (**troubleshooting**); gather feedback (**sounding board**); share information, experiences, and resources (**sharing experience**); provide encouragement (**affective support**); and explore concepts related to their domain of practice (**explore pedagogical concept**). Without the FOLC, these opportunities may be limited or less relevant, especially if faculty are the only person in their department teaching this course. Here, we provide evidence of the formation of a Next Gen PET community and the role of community in achieving the desired outcomes for FOLC participants, specifically through the mediating processes the community affords.

**Fig. 2:**
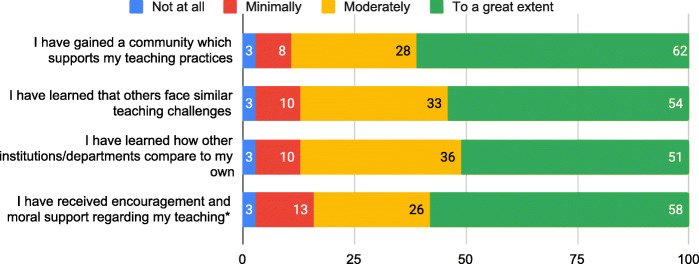
Community. Percent of respondents reporting benefits to participating in the FOLC in spring 2019 (end of year 2 of the FOLC). Participants rated the extent to which each benefit had occurred as a result of their participation in the FOLC. Items were not presented to participants in this order or identified with the category *community*. Numbers superimposed on the bars are the percentage of respondents choosing that option. (*N* = 39, except for *. *N* = 38 for * items)

Because a FOLC is an example of a CoP, it is essential to establish a strong sense of community between FOLC members (Wenger et al., 2002). Therefore, we contend that there cannot be an effective FOLC without a sense of community established between members. In spring 2019, participants were surveyed about the benefits of participating in the FOLC. Four potential benefits related to the FOLC as a community that supported participants. (See the “Data sources and analysis” section for how the benefits were categorized.) The results on these items (see Fig. 2) strongly indicate that many Next Gen PET FOLC members feel they are part of a community. Results include the responses from both the novice Next Gen PET instructors and the cluster leaders. Nearly all respondents (90%) agreed (moderately or to a great extent) that they have gained a community which supports their teaching due to their participation in the FOLC. The distributed nature of the community and online interactions did not preclude forming a community; on the contrary, it allowed participation in a highly relevant community by faculty who may not otherwise have colleagues to discuss this course with. In response to an open-ended question on the spring 2019 survey about what they like most about FOLC meetings, one participant reported, “Getting to talk to other physics teachers who are like minded. My own department is small and my colleagues have somewhat different goals and ideas than I do. Being able to talk to both populations is valuable.” Through the FOLC, this participant had access to a group of faculty who held similar teaching goals, a community they were missing at their local institution.

Figure 2 also shows that 84% of respondents agreed (moderately or to a great extent) that they had received encouragement and moral support regarding their teaching because of their FOLC participation. The FOLC was a source of **affective support** for most respondents. There is evidence of *how this mediating process leads to outcomes* in the response of one participant to a survey question about the most significant impact of participating in the FOLC. This participant shared, “I enjoy the camaraderie. I especially enjoy the support and encouragement from my FOLC group. The meetings really motivate me to do better, and to be accountable for my teaching and students.” Affective support (e.g., encouragement) can act to *motivate* participants to continually improve their instruction and additionally lead to a productive sense of accountability regarding their teaching practice (Corrales et al., 2020; Dancy et al., 2019).

Because faculty were meeting with others from different institutions, they gained an awareness of expectations for course content coverage and student outcomes at other institutions. This mediating process of **sharing experiences** provided a valuable calibrating function to faculty trying to gauge their expectations for student performance. For example, in response to the open-ended question about the most significant impact of the FOLC, one participant reported, “I have benefited from having a community of people using the same curriculum that I am using because... [it has] allowed me to hear how other students are doing so I don’t have unreasonable expectations for my own students.” More broadly, participants gained knowledge of what other institutions are like and how they function, such as different course structures or teacher preparation models. The vast majority of responding participants reported that they learned how other institutions/departments compared to their own (87% responded “moderately” or “to a great extent”), and that others face similar teaching challenges (here also 87% responded “moderately” or “to a great extent”). In the words of one participant, “It has been useful to find out how challenges are handled by other faculty at other institutions.” These comments indicate that it is exactly through participating in this community of Next Gen PET adopters that participants receive useful knowledge about student performance in the course and ideas for how to solve common challenges (i.e., troubleshooting), thus highlighting the community’s role as a mechanism for supporting faculty members’ implementations of the curriculum.

Results from one of the open-ended survey questions show that not only is a community established, but participants highly value the FOLC community. The question asked participants what they found most valuable about their participation in the FOLC. Almost all of the 33 responses cited sharing knowledge (i.e., experience)/implementation support (i.e., troubleshooting) and/or the community they formed with their FOLC members as the most valuable part of the FOLC experience. Often, the value of sharing knowledge and the community were intertwined. For example, one FOLC member said, “It is nice to talk with people who have approached the same problems from different perspectives, and so have developed solutions I might not have thought of myself.” For this member, the implementation help and knowledge they gained was particularly valuable because it came from people who are teaching Next Gen PET and who have different experiences and ideas than the respondent. Another participant echoed this idea, sharing, “Sometimes it is just nice to talk with other people about how your class is going and it is really helpful when the other people understand what you're trying to accomplish and why you are teaching in a certain way.” Participants value the opportunity to connect with instructors who have similar pedagogical alignments and are invested in implementing Next Gen PET. The Next Gen PET FOLC provides this community for instructors.

The survey responses that demonstrate the overlapping influences of community and idea sharing also provide evidence that the community offers a **sounding board** for ideas. For example, one FOLC participant shared that they most valued, “Getting to toss around ideas about teaching with other like-minded teachers,” and someone else responded about valuing, “The ability to loft a question out there, big or small, and get supportive feedback.” The “tossing around ideas” and “lofting a question out there” suggest that the community welcomed the casual exchange of ideas and there was a supportive back-and-forth around those shared suggestions.

We see from these results that FOLC members do feel a sense of community in the FOLC. This community provides encouragement and affective support through teaching challenges for its members. It provides the additional mediating processes of a sounding board for ideas, a space to share experiences, and a venue for troubleshooting.

Together, the specific example provided by the meeting transcript, along with the broader perspectives gathered by the survey, demonstrate how these mediating processes are provided in the Next Gen PET FOLC and how they serve as mechanisms faculty can use to meet desired FOLC outcomes. By connecting instructors with shared knowledge of the curriculum and similar pedagogical values and beliefs, the FOLC is uniquely positioned to provide *specific* implementation advice and pedagogical knowledge to participants, information that can either be directly implemented or that motivates further reflection around members’ teaching practices. These are desired outcomes for Next Gen PET FOLC members.

### Results part 2: outcomes

We now address our second research question: *If the FOLC provides opportunities for the mediating processes to occur*, *to what extent are the outcomes listed in the conjecture map* (Fig. 1) *achieved*? More specifically, 2 years into the lifetime of the FOLC, to what extent are the proximal outcomes achieved, namely, familiarity with Next Gen PET structure, content, and materials (familiarity); increased knowledge of pedagogical techniques (knowledge); increased confidence in using curriculum (confidence); reflection on Next Gen PET teaching practice (Next Gen PET reflection); and expanded use of pedagogical techniques aligned with Next Gen PET core principles (Next Gen PET techniques)? The results presented in this section come from the spring 2019 survey (administered at the end of year 2 of the FOLC). Given the timing of the data collected and the fact that the FOLC is currently still running, we can only speak to indications of achieving the longer-term goals for FOLC participants. The more distal outcomes include student learning gains in the Next Gen PET course (student learning), persistence in using the curriculum (persistence), reflection in teaching practice across courses taught (general reflection), and use of research-based pedagogy in other courses (ripple effects). This section focuses on achievement of the proximal outcomes, but will present early results regarding distal outcomes where possible.

In this section, we first focus on factors directly related to implementing the Next Gen PET curriculum. Then we describe broader impacts on participants’ teaching that could extend beyond the Next Gen PET course. Results include the survey responses from both the novice Next Gen PET instructors and the cluster leaders.

### Preparedness to implement the curriculum

**Fig. 3:**
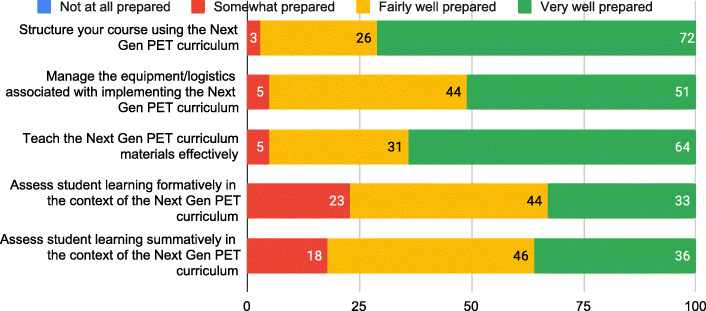
Preparedness. Faculty’s sense of preparedness regarding the Next Gen PET curriculum, in spring 2019 (end of year 2 of the FOLC). *N* = 39. Respondents were asked to “Indicate your current level of preparedness to do each of the following” using a four-point scale: Not at all prepared; Somewhat prepared; Fairly well prepared; or Very well prepared. Numbers superimposed on the bars are the percentage of respondents choosing that option. No respondents selected “Not at all prepared” on any of these items

On the spring 2019 survey, FOLC members were asked about their preparedness to do a range of things with the curriculum. The results are included in Fig. 3. Overall, at this time point, respondents felt prepared to implement the curriculum (structuring, logistics, teaching), with at least 95% of respondents feeling “fairly well” or “very well” prepared in those categories. In regard to assessing student learning, all respondents felt at least “somewhat prepared,” and the majority (over 77%) reported feeling “fairly well” or “very well” prepared to do so. The same questions were asked of participants immediately after the initial in-person workshop (in summer 2017, prior to the FOLC’s start). At that time, participants were asked about their preparation to implement the Next Gen PET curriculum before the workshop (retrospective pre-workshop) and after the workshop. The percent of faculty reporting a sense of preparedness at the levels of “fairly well” or “very well” prepared increased at all three time points (Smith & Wingard, 2019), indicating positive growth (see Supplemental Material for detailed statistics). While this is perhaps not surprising, as participants had more and more experience with the curriculum at each time point, it confirms a trend we hoped to see.

**Fig. 4:**
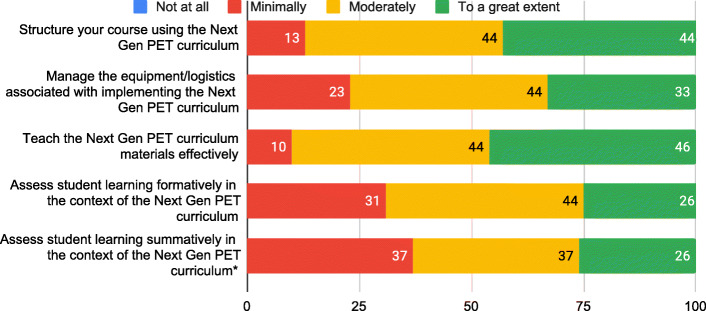
Preparedness due to FOLC participation. Faculty’s sense of the extent to which *participating in the FOLC* has prepared them to use the Next Gen PET curriculum, as of spring 2019 (end of year 2 of the FOLC). *N* = 39, except for * item where *N* = 38. Respondents were asked to rate “To what extent has participating in the FOLC prepared you to do each of the following” on a four-point scale: Not at all; Minimally; Moderately; or To a great extent. Numbers superimposed on the bars are the percentage of respondents choosing that option. No respondents selected “Not at all” on any of these items

Feelings of preparedness to implement the curriculum could be due to a number of factors, starting with simply having taught the course one or more times. In order to evaluate how participating in the FOLC contributed to a sense of preparedness, the spring 2019 survey also asked participants to what extent had *participating in the FOLC* prepared them to teach their Next Gen PET courses. Results from this question are shown in Fig. 4. Between 77 and 90% of respondents reported that the FOLC moderately or to a great extent prepared them to teach Next Gen PET effectively, handle logistics, and structure their courses. Fewer, but still a majority, felt the FOLC prepared them moderately or to a great extent to assess student learning. Evaluation of student outcomes is typically a later concern than course management (Gene, George, & Stiegelbauer, 2013), and respondents’ relatively lower sense of preparation to assess student learning may reflect that.

The questions on preparedness to implement Next Gen PET speak to a number of the immediate goals of the FOLC. The responses to these questions tell us most directly about the goals that participants will increase their familiarity with the Next Gen PET structure, content, and materials (familiarity goal), and their confidence in using the curriculum (confidence goal)^7^. The high levels of preparedness respondents report regarding their ability to structure the Next Gen PET course, handle logistics, and teach with the materials effectively, and the increase in the percent of participants indicating “fairly well prepared” or “very well prepared” since the initial in-person workshop are indications that participants are meeting the familiarity goal. Figure 4 provides evidence that the FOLC specifically is contributing to participants’ familiarity with the materials. In addition, these sense-of-preparedness items can be indications of confidence in using the curriculum (confidence goal). That is, one expects an increased sense of preparedness to result in increased confidence. Combining responses on all 5 preparedness statements into a composite score^8^ and then testing how the composite scores compare across time points, we find that there is a significant increase in scores at the later times. (See Table S1 in the Supplemental material for details on the statistical analysis performed.) The spring 2019 preparedness composite scores were on average 1.61 standard deviations higher (large effect size (Cohen, 1988)) than the scores at the retrospective pre-workshop time point (Hierarchical Linear Model, HLM, *t*(35) = − 10.26, *p* < 0.05) (Raudenbush & Bryk, 2002) and on average 0.79 standard deviations higher (medium effect size) than the scores at the post-workshop time point (HLM, *t*(35) = − 4.68, *p* < 0.05) (Smith & Wingard, 2019). We also see that the majority of respondents say *the FOLC* moderately or to a great extent prepared them in these areas.

### Benefits of participation in the FOLC

The spring 2019 survey also asked participants about the benefits resulting from participating in the FOLC. Participants were asked to what extent they had experienced 15 potential benefits due to their participation in the FOLC; the list of potential benefits was developed and analyzed as described in the “Methods” section. Below, we report on the results from this benefit question, presenting the items sorted into five categories based on post-hoc grouping: Teaching practice & pedagogy; Attitude toward teaching; Student impact; Time & efficiency; and Community. (The four benefit items relating to the FOLC community category are reported above, in Results part 1.)

### Participants’ perceptions of FOLC impact on their teaching practice and pedagogy

**Fig. 5:**
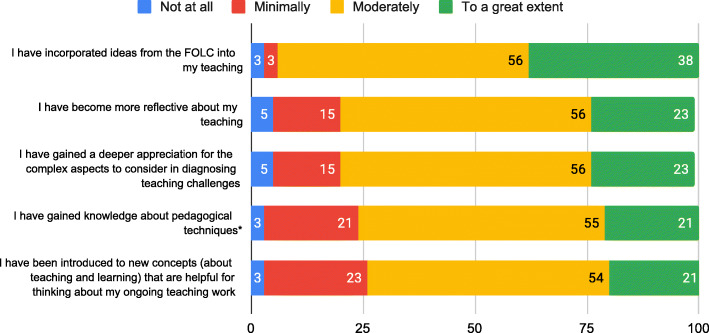
Teaching practice and pedagogy. Percentage of respondents reporting benefits to participating in the FOLC in spring 2019 (end of year 2 of the FOLC). Participants rated the extent to which each benefit had occurred as a result of their participation in the FOLC. Items were not presented to participants in this order or identified with the category *Teaching practice and pedagogy*. (*N* = 39, except for *. *N* = 38 for * items)

Increased knowledge of pedagogical techniques (knowledge goal) and expanded use of pedagogical techniques aligned with the Next Gen PET core principles (Next Gen PET techniques goal) were important anticipated outcomes from FOLC participation, and indeed, participants widely attributed changes in teaching practice to their participation in the FOLC (see Fig. 5). The benefits listed in Fig. 5 include gaining knowledge of pedagogical *techniques* as well as pedagogical *concepts*, and putting these ideas into action through their teaching and reflection on their practice. Illustrating these benefits, when asked in an open-ended format about the most significant impact of participating in the FOLC; one member shared “Seeing and experiencing the implementation of new teaching methods that are vastly different than what I’ve done before. This has opened the possibilities of the classroom like never before for me.” This participant has expanded their pedagogical toolkit in learning about active learning teaching techniques that are “vastly different” from what they are used to doing. Another participant shared that the most significant impact for them was that they “have become more interested in learning more about how people learn, and I’ve started talking with people over in our college of education.” This participant was inspired to educate themselves more about learning and pedagogy.

The teaching practice and pedagogy items also included statements about participants’ reflective practice, another expected outcome. Improved teaching reflection can have long-lasting and far-reaching impact by providing a process for ongoing development (Rodgers, 2002). As one respondent reported, “The most significant impacts of participating in the FOLC is participating in a community that supports my teaching practices, as well as being more reflective in my practices as I work with my colleagues and others in the FOLC.”

The benefits to teaching practice and pedagogy reported by participants indicate that the FOLC is meeting a number of its goals. This data suggest that we are meeting the immediate-term goals of increasing participants’ knowledge of pedagogical techniques (knowledge goal), reflection on their teaching practice in the Next Gen PET course (Next Gen PET reflection goal), and use of pedagogical techniques aligned with the Next Gen PET core principles (Next Gen PET techniques goal). Moreover, the teaching practice and pedagogy items do not exclusively refer to the Next Gen PET course so they also speak to our longer-term goals for participants of applying research-based pedagogical techniques to their other courses (ripple effects goal) and engaging in reflection on their teaching practice in all their courses (general reflection goal). Faculty have gained a deeper appreciation for the complex aspects to consider in diagnosing teaching challenges, pedagogical knowledge, and reflection skills, all of which can be applied across their teaching practice.

### Participants’ perceptions of FOLC impact on their attitudes toward teaching

**Fig. 6:**
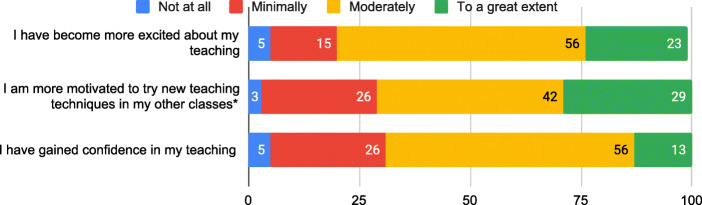
Attitude toward teaching. Percentage of respondents reporting benefits to participating in the FOLC in spring 2019 (end of year two of the FOLC). Participants rated the extent to which each benefit had occurred as a result of their participation in the FOLC. Items were not presented to participants in this order or identified with the category *attitude toward teaching*. (*N* = 39, except for *. *N* = 38 for * items)

In this section, we focus on motivation, enthusiasm, and confidence, which we categorize as attitudes toward teaching. The FOLC contributed positively to its members’ attitudes toward teaching. As shown in Fig. 6, over two-thirds of respondents reported that the FOLC moderately or to a great extent contributed to increased motivation, excitement, and confidence in their teaching. One FOLC member relayed that the most valuable aspect of their FOLC involvement was, “Again, being able to get advice and compare notes, and finding that others found value in some of my ideas.” It is reasonable to conclude that this member gained some confidence in their teaching given that their fellow FOLC members found their ideas useful.

This set of benefits directly relates to the immediate-term Next Gen PET FOLC goal that participants will increase their confidence in using the curriculum (confidence goal) and the longer-term goal that participants will use research-based pedagogy in their other courses (ripple effects goal). It is particularly encouraging to see that 71% of respondents say that they are motivated moderately or to a great extent by their FOLC participation to try new teaching techniques in their non-Next Gen PET courses. Even at the end of year 2 of the Next Gen PET FOLC, there are indicators that participation will have an impact beyond members’ Next Gen PET course.

### Participants’ perceptions of FOLC impact on their students

**Fig. 7:**
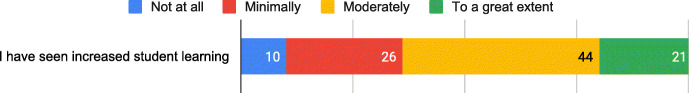
Student impact. Percentage of respondents reporting benefits to participating in the FOLC in spring 2019 (end of year 2 of the FOLC). Participants rated the extent to which each benefit had occurred as a result of their participation in the FOLC. Items were not presented to participants in this order or identified with the category *student impact*. (*N* = 39)

The immediate goal of the FOLC is to support faculty in implementing an effective, research-based curriculum. Next Gen PET has been shown to lead to student learning gains on a conceptual assessment of the physical science material (Engelhardt et al., 2018; Smith & Wingard, 2019), but it is important to recognize the complex, nonlinear nature of the connection between curriculum, faculty implementation, and student outcomes (Manduca, 2017). Implementing research-based teaching strategies is messy work that typically requires tailoring instructional practices to a local context (student population, course format, programmatic or departmental expectations, etc.). Despite all of this, nearly two-thirds of respondents reported that they have moderately or to a great extent seen increased student learning as a result of their participation in the FOLC, as indicated in Fig. 7. While we do not have further information on the basis for participants’ reports of increased student learning, we consider this an encouraging indicator, especially considering that the Next Gen PET curriculum was new to most FOLC members at the start of the FOLC. Of course, it is hard to disentangle the impact of the FOLC from the impact of the curriculum on student learning as the instructors’ experiences of teaching the curriculum and participating in the FOLC are intertwined. Nevertheless, the fact that the majority of respondents report seeing increased student learning shows progress on the long-term goal of achieving student learning gains in the Next Gen PET course (student learning goal). It also likely increases the chance that faculty will continue using Next Gen PET (persistence goal).

It is also worth noting that we have heard of (unexpected) learning opportunities for students due to their instructor’s participation in the FOLC. One survey respondent said that, “One of the most useful aspects of FOLC was to let my students know that I was discussing issues about how the course was being presented so that provided them with a greater understanding of the teaching process, and the students appreciated that.” Recall, many of the students taking the Next Gen PET course are prospective elementary teachers and they have more to learn than the content contained in the Next Gen PET curriculum; they are also learning about the practice of teaching.

### Participants’ perceptions of FOLC impact on their time and efficiency

Participating in the FOLC takes time, and this may be a significant deterrent to faculty involvement. Faculty commonly cite lack of time as preventing them from implementing research-based teaching techniques (Dancy & Henderson, 2010; Turpen, Dancy, & Henderson, 2016). It is therefore important that any program to support faculty in implementing these techniques respect the time of faculty who are often overworked and balancing multiple responsibilities. Hopefully, the benefits to faculty from participating in a FOLC motivate and justify the investment of time, or even save time in their teaching development overall. This can happen, for instance, if the FOLC provides help and resources that reduce the time required to prepare, locate resources, or develop relevant teaching skills.

**Fig. 8:**
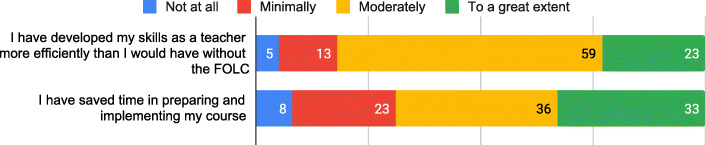
Time and efficiency. Percentage of respondents reporting benefits to participating in the FOLC in spring 2019 (end of year 2 of the FOLC). Participants rated the extent to which each benefit had occurred as a result of their participation in the FOLC. Items were not presented to participants in this order or identified with the category *time and efficiency*. (*N* = 39)

It is therefore positive that the majority of respondents (82%) reported that they moderately or to a great extent developed their skills as a teacher more efficiently than they would have without the FOLC, and 69% of respondents said participating in the FOLC moderately or to a great extent contributed to them saving time preparing and implementing their course (see Fig. 8). In responding to the open-ended question on the most significant impact of participating in the FOLC, one participant said that, “having a network of people makes it very efficient to get started [on a new teaching prep].” Implementing a new course can be an immense undertaking, but this participant recognized the FOLC as lessening that burden. Another response to the significant impact question offers perhaps one reason why the FOLC helps some participants save time in their teaching work; this respondent said, “[The FOLC] enabled me to anticipate difficulties that students and instructors have with the curriculum, enabling me to be more prepared in my own teaching.” The implementation experiences shared in the FOLC helped this member feel more prepared in teaching their course. These time and efficiency statements do not directly relate to the Next Gen PET FOLC goals for participants, but they suggest that members find participation in the FOLC to be a worthwhile investment of time. This is important feedback for the Next Gen PET FOLC designers that the program is not an onerous commitment for many participants, and that participants are experiencing the benefits of efficiency and time savings.

### Impact on other courses

**Fig. 9:**
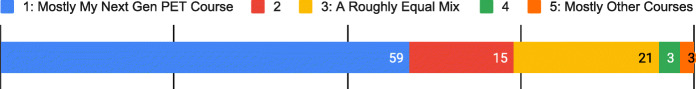
Courses impacted by participation in the FOLC. The effect of FOLC participation on the range of courses members teach. Respondents indicated if the FOLC impacted mostly their teaching of the Next Gen PET course, mostly the other courses they teach, or a mix of both. Numbers superimposed on the bar are the percentage of respondents choosing that option. *N* = 39

In order to determine if participating in the FOLC is impacting members’ teaching in their non-Next Gen PET courses, we included a question on the spring 2019 survey that asked “Has participating in the FOLC impacted mostly your teaching of the Next Gen PET course, mostly the other courses you teach, or a mix of both?” Participants responded on a five-point scale ranging from “Mostly my Next Gen PET course” to “Mostly other courses.” The results from this question are displayed in Fig. 9. Unsurprisingly, the majority (59%) reported impacts primarily on their Next Gen PET course, but encouragingly 41% indicated the FOLC had some impact on their teaching of other courses as well. We do not expect to see much transfer to other courses at the end of year 2 of the FOLC, especially since the focus of the first 2 years was heavily on implementing the Next Gen PET course, so these results are promising for the potential impact participating in the FOLC will have on members’ teaching overall at the end of the 4 years. Even at the 2-year mark, 21% of respondents said that participating in the FOLC has impacted “A roughly equal mix” of their Next Gen PET and non-Next Gen PET courses. In answering the open-ended question about the most significant impact of the FOLC, one participant shared, “I have learned new methods of presenting certain ideas that I now use in my traditional conceptual physics course.” The “ripple effect” of impact to other courses is related to two of the long-term outcomes we hope FOLC participants achieve: using research-based pedagogical techniques in their other courses (ripple effects goal) and engaging in reflection on their teaching practice in these other courses (general reflection goal). The results from the question asked on the spring 2019 survey provide early indications of participants meeting these goals. In the future, we plan to collect more information about the range of courses members are applying their learning to and what exactly they are transferring.

### Summary of outcomes

The survey results presented here indicate that, to a large extent, the FOLC is meeting its intended outcomes for participants (i.e., its goals for participants). Specifically, participants report increased confidence in using the curriculum (confidence goal); familiarity with Next Gen PET structure, content, and materials (familiarity goal); increased knowledge of pedagogical techniques (knowledge goal); reflection on teaching practices in the Next Gen PET course (Next Gen PET reflection goal); and expanded use of pedagogical techniques aligned with the Next Gen PET core principles (Next Gen PET techniques goal). There is also emerging evidence related to more distal outcomes, including student learning gains in the Next Gen PET course (student learning goal), persistence in using the curriculum (persistence goal), reflection in teaching practice across courses taught (general reflection goal), and use of research-based pedagogy in other courses (ripple effects goal). Quotes from participants’ open-ended responses to questions about the most significant impact of FOLC participation as well as the most valuable aspect of the FOLC provided further insight into the nature of these outcomes for individual participants. As a summary of the information presented above, Table 4 compiles the survey items that provide support for each FOLC outcome.

**Table 4:** Summary of survey results showing the Next Gen PET FOLC outcomes are being met

Next Gen PET FOLC goals for participants	Survey items whose results indicate goal is being met	Main findings
Immediate-term goals		
Increased confidence in using the curriculum	Preparedness to teach Next Gen PET (Figs. 3 and 4) Attitude toward Teaching Impacts (Fig. 6)	Faculty report being very well prepared to teach Next Gen PET and over two-thirds report moderately or greatly increased confidence in their teaching
Increased familiarity with Next Gen PET structure, content, and materials	Preparedness to teach Next Gen PET (Figs. 3 and 4)	Faculty report that, due to participation in the FOLC, they are prepared to structure, manage, and teach their Next Gen PET courses effectively.
Increased knowledge of pedagogical techniques	Teaching Practice & Pedagogy Impacts (Fig. 5)	Faculty report having learned pedagogical techniques and concepts
Increased reflection on teaching practices in the Next Gen PET course	Teaching Practice & Pedagogy Impacts (Fig. 5)	Faculty report becoming more reflective, and gaining a deeper appreciation of the complexity of diagnosing teaching challenges
Expanded use of pedagogical techniques aligned with the Next Gen PET core principles	Teaching Practice & Pedagogy Impacts (Fig. 5)	Faculty report having incorporated ideas from the FOLC into their teaching and having been introduced to new concepts.
Longer-term goals		
Student learning gains in the Next Gen PET course	Student Impacts (Fig. 7)	Some emerging evidence for increased learning, based on faculty reports
Persistence in using the curriculum^a^	Student Impacts (Fig. 7)	Members of the FOLC have continued using the Next Gen PET curriculum
Reflection in teaching practice across courses taught	Teaching Practice & Pedagogy Impacts (Fig. 5) Impact on Other Courses (Fig. 9)	Faculty report becoming more reflective about their teaching; one-fifth of faculty report a roughly equal impact on their Next Gen PET and other courses.
Use of research-based pedagogy in other courses	Teaching Practice & Pedagogy Impacts (Fig. 5) Attitude toward Teaching Impacts (Fig. 6) Impact on Other Courses (Fig. 9)	Faculty report benefits that are not specific to Next Gen PET courses and being motivated to try new techniques in other classes. One-fifth of faculty report a roughly equal impact on their Next Gen PET and other courses.

^a^Note, for the “Persistence in using the curriculum” outcome, continuing participation in the FOLC is additional evidence of members still teaching the curriculum, as use of the Next Gen PET curriculum is an expectation for FOLC membership

## Discussion and future directions

The research presented in this article responds to a gap in the literature regarding effective support mechanisms for adopters of educational innovations, specifically research-based instructional strategies (Khatri et al., 2016). In particular, we studied a faculty online learning community (FOLC) as a model of faculty support. A FOLC is a professional community of faculty dedicated to improving their teaching practice; they meet regularly via online video conference to talk about their teaching and connect on a chat platform in between meetings. Unlike one-off presentations or multi-day workshops, FOLCs establish a long-term (year or longer) community that runs contemporaneously with participants’ in-class implementations of new teaching strategies. The site of our study was a FOLC for adopters of the Next Gen PET curriculum. The curriculum requires instructors to implement both non-traditional content and pedagogy into their physical science course. Due to these characteristics of the curriculum, we hypothesized that adopters would benefit from long-term, people-based implementation support in the form of a FOLC.

The Next Gen PET FOLC was designed to provide opportunities for participating faculty to share their ideas and experiences; troubleshoot teaching challenges; provide and receive affective support; have a sounding board for ideas; and explore pedagogical concepts in the context of problems of classroom practice. In Results part 1, a sample meeting transcript and survey data demonstrated the enactment and prevalence of these mediating processes in the Next Gen PET FOLC, as well as how these processes are linked to desired outcomes. For example, these processes can motivate participants to make a change in their teaching practice or reflect on their Next Gen PET implementation. In Results part 2, survey data demonstrated that the majority of FOLC participants are achieving the short-term goals of the program, and there are early indications of long-term goals being achieved. Together, these results indicate that the mediating processes present in the FOLC are *contributing* to the outcomes for participants^9^. In turn, this speaks to the efficacy of the FOLC model in supporting Next Gen PET adopters.

It is reasonable to expect that for similarly demanding educational innovations, the FOLC could be a productive means for supporting adopters. That is, FOLCs could productively support adopters of other innovations that require an instructor to implement non-traditional pedagogy and content coverage, make decisions about materials and format, and perhaps additionally acquire new material and personnel resources. We expect that the mediating processes in the Next Gen PET FOLC (e.g., sounding board for ideas; affective support) could also help adopters of different, demanding educational innovations achieve comparable outcomes (e.g., success in implementing the new innovation and reflecting on their implementation).

Future work can help determine the general utility of the FOLC model for supporting different faculty populations and their adoption of a range of teaching strategies and materials. For example, future research should explore if FOLCs are useful for adopters of innovations that require only a modest level of change from current practice on the part of the adopter. If an educational innovation perhaps only required instructors to make a small change to the content they cover in their class, would those adopters benefit from a FOLC group established around that innovation, or would the degree of change be too small to sustain discussions in a long-term community? Additionally, most faculty in this FOLC were teaching courses for future elementary teachers; this population may be uniquely motivated to implement active learning teaching styles. Would FOLCs involving other faculty populations see similar results?

It also remains to be seen if the mediating processes in the Next Gen PET FOLC can contribute to outcomes beyond those documented in the Next Gen PET case. For example, can opportunities for troubleshooting and a sounding board for ideas help community members successfully navigate their local institutional dynamics in order to garner the resources they need to implement an innovation, or perhaps even gather buy-in from their colleagues or entire department? It seems plausible that the FOLC could help members gain these skills, but the Next Gen PET FOLC data presented here does not directly speak to that outcome.

Similarly, future work should explore if additional or a distinct set of mediating processes could equivalently contribute to the outcomes Next Gen PET members are achieving. We have argued that the mediating processes in the Next Gen PET FOLC are at least one factor contributing to the outcomes for FOLC members, but we cannot say if these are *necessary* processes or simply *sufficient* processes. Future research should examine this question of necessary processes. For example, what outcomes would be observed if the main mediating process offered by a FOLC was hearing lectures from experts in educational innovations?

In addition to studying the possible types of mediating processes, future work should also explore how the processes can be enacted. In the Next Gen PET FOLC, the mediating processes are enacted through conversations during the video conference meetings and on the chat platform for the community. The impact of meeting structure and facilitation practices on the nature of the meetings is an area of ongoing work. In addition, there are potentially other effective ways to enact the mediating processes. For example, a FOLC group could hold a quarterly, community-wide virtual conference that offers a venue for participants to formally share their experiences and resources. Alternatively, a FOLC could employ a structured set of activities to elicit participants’ experiences and questions. Further information on successful enactments of mediating processes can help guide design decisions for future FOLCs.

As we finalized this manuscript, the COVID-19 pandemic had caused a sudden, unplanned transition to online instruction for the faculty in the Next Gen PET FOLC. The need to work remotely and practice social distancing highlighted the affordances of an *online* model of professional development. Discussion of the transition to online instruction dominated Zoom meetings and Slack postings during spring 2020, as faculty shared ideas, resources, and tools. The FOLC clearly helped instructors transition their courses online, but describing and analyzing the FOLC’s role in supporting faculty during this time is beyond the scope of this paper. This will be a focus of future work.

## Conclusion

The propagation paradigm (Khatri et al., 2016) calls attention to the need for ongoing support for adopters of RBISs. The FOLC model provides participating faculty with ongoing support through participation in a community. The Next Gen PET FOLC is an effective people-based support mechanism for adopters of the Next Gen PET curriculum. Next Gen PET FOLC members are increasing their knowledge of pedagogical techniques, expanding their use of pedagogical techniques aligned with the Next Gen PET core principles, and beginning to spread these practices to their other courses. This is facilitated by the opportunities in the FOLC for troubleshooting, idea sharing, and receiving encouragement through challenges. We anticipate that this FOLC model will work to support adopters of educational innovations that are demanding in terms of both content and pedagogy changes and choices instructors must make. Future work will help determine the scope of the applicability of FOLCs as a support mechanism for adopters of an educational innovation.

## Supplementary information

Additional file 1: **Table S1**. Participants’ Perceptions of Preparedness^†^ for Various Aspects of Implementing Next Gen PET. **Table S2**. Raw Composite Scores.

## Data Availability

Curriculum available at https://nextgenpet.activatelearning.com. Survey data and meeting transcript files are not publicly available due to confidentiality concerns.

## Abbreviations

FOLC: Faculty online learning community
Next Gen PET: Next Generation Physical Science and Everyday Thinking
RBIS: Research-based instructional strategy
CoP: Community of practice
FLC: Faculty learning community

## Footnotes

1 It should be noted that often the students studied in physics education research are unrepresentative of the total population of physics students in the USA, leaving the generalizability of the results from these studies suspect (Kanim & Cid, 2020).

2 While this paper describes work that occurred before COVID-19, the model of faculty support studied here is highly relevant to faculty needs during the pandemic. We touch on this in the discussion.

3 As William Sandoval describes, conjecture mapping is a method used in educational design research for “specifying theoretically salient features of a learning environment design and mapping out how they are predicted to work together to produce desired outcomes” (p. 19, Sandoval 2014). The map thus directs one’s empirical observations and specifies connections to be tested between design elements, processes, and outcomes. In this paper, we adapt Sandoval’s conjecture mapping, focusing specifically on mediating processes, outcomes, and the connection between the two (posed as a “theoretical conjecture” (Sandoval’s terminology)).

4 We use the term “novice Next Gen PET instructors” only to refer to instructors’ experience with the Next Gen PET curriculum and to distinguish this group from the cluster leaders, who had experience with Next Gen PET. We do not mean to suggest these faculty were novice instructors. Many of the novice Next Gen PET instructors had experience with prior versions of the Next Gen PET curriculum, or with other guided-inquiry approaches. More information on their background is included in the “Participant demographics” section.

5 Groups that are underrepresented in the physical sciences must contend with microaggressions (Sue, 2010), implicit biases (Schmader, Whitehead, & Wysocki, 2007), and stereotype threat (Steele & Aronson, 1995). This all affects their experiences in science. For example, STEM students who identify with the LGBTQ+ community contend with unique challenges in active learning classrooms (Ackerman et al., 2018; Cooper & Brownell, 2016).

6 We acknowledge this as a limitation of our study. An important area of future work will be to disaggregate the experiences of faculty members from different demographic groups in a FOLC to explore if the community is supportive and productive for all members.

7 Note, the preparedness results tell us most clearly about the familiarity and confidence goals, but they also could indicate meeting some of our other goals such as increased knowledge and use of pedagogical techniques. For example, feeling prepared to teach the curricular materials effectively likely have to do both with the mechanics of being familiar with the materials as well as feeling knowledgeable about the pedagogy utilized with the curriculum.

8 The composite score for preparedness was calculated by summing the responses to the items in Fig. 3 and then dividing by the total points possible. To put the composite on a 100-point scale, the lowest response option was set to 0, and the others were adjusted accordingly. The preparedness response scale, which originally ranged from 1 to 4, was recoded to have a scale of 0 to 3. The denominator was determined by computing the maximum possible sum of responses for the series of items. A respondent’s score was then calculated as (sum of responses/maximum possible sum)x100.

9 Of course, participants’ experiences teaching the curriculum, as well as potentially other factors, may contribute to the achievement of these outcomes as well.
