# Supplementary material for: Analyzing a faculty online learning community as a mechanism for supporting faculty implementation of a guided-inquiry curriculum
Source: Int J STEM Educ. 2021 Feb 24;8(1):17. doi: 10.1186/s40594-020-00268-7 (PMC7902091; doi:10.1186/s40594-020-00268-7)
Supplement: Supplementary file 1 — Table S1. Participants’ Perceptions of Preparedness† for Various Aspects of Implementing Next Gen PET. Table S2. Raw Composite Scores. [file 40594_2020_268_MOESM1_ESM.docx]

# Supplemental Material

**Spring 2019 Survey**

**NGP FOLC Feedback 2018–19**

1. To what extent has each of the following occurred as a result of participating in the FOLC during the 2018-19 academic year: [not at all, minimally, moderately, to a great extent]
   1. I have become more reflective about my teaching.
   2. I have gained confidence in my teaching.
   3. I have gained knowledge about pedagogical techniques.
   4. I have learned that others face similar teaching challenges.
   5. I have learned how other institutions/departments compare to my own.
   6. I have developed my skills as a teacher more efficiently than I would have without the FOLC.
   7. I have saved time in preparing and implementing my course.
   8. I have seen increased student learning.
   9. I have incorporated ideas from the FOLC into my teaching.
   10. I have gained a community which supports my teaching practices.
   11. I have received encouragement and moral support regarding my teaching.
   12. I have become more excited about my teaching.
   13. I am more motivated to try new teaching techniques in my other classes.
   14. I have been introduced to new concepts (about teaching and learning) that are helpful for thinking about my ongoing teaching work.
   15. I have gained a deeper appreciation for the complex aspects to consider in diagnosing teaching challenges.
   16. Other; please specify*
2. Please briefly describe the most significant impact(s) of participating in the FOLC. Please feel free to include impacts that are not listed in Question 1. [text box]
3. Has participating in the FOLC impacted mostly your teaching of the Next Gen PET course, mostly the other courses you teach, or a mix of both?

Mostly my Next A roughly Mostly other

Gen PET course equal mix courses

􀀀 􀀀 􀀀 􀀀 􀀀

Please use the space below to elaborate if you wish:

1. How would you describe the balance of attention to practical issues (e.g., pacing, equipment) and pedagogical issues (e.g. student learning, promoting student engagement) in the FOLC?

Too much The right balance Too much

attention to between practical attention to

practical issues and pedagogical pedagogical

issues issues

􀀀 􀀀 􀀀 􀀀 􀀀

1. What did you find most valuable about your involvement in the FOLC? [text box]

**Cluster Feedback**

1. How often have you participated in cluster meetings during the 2018-19 academic year? (Most clusters scheduled one meeting per month this year; cluster 5 met more often.)

Never About half Every time

the time

􀀀 􀀀 􀀀 􀀀 􀀀

1. What do you like most about cluster meetings? [text box] [skip if Q6 = never]
2. What could be better about cluster meetings? [text box] [skip if Q6 = never]
3. Did you participate in a project group?

○ Yes

○ No

○ Sort of/not sure

**Project Group Feedback**

1. What interested you in the project you are involved in? Select all that apply [Display only if Q9=Yes]

❏ I am interested in the topic for my teaching.

❏ The topic ties in with other interests I have (research, previous work, etc).

❏ I wanted to work with other people who were involved.

❏ Other; please specify*

1. How satisfied are you with the project you are involved in? [Display only if Q9=Yes]

Not Satisfied Minimally Satisfied Moderately Satisfied Very Satisfied

1. What do you like about being involved in the project? [Display only if Q9=Yes]
2. What could the Next Gen PET FOLC do to improve being involved in the project? [Display only if Q9=Yes]
3. To what extent did each of the following limit your involvement in a project group? [not at all, minimally, moderately, to a great extent]
   1. lack of time
   2. lack of interest
   3. scheduling difficulties
   4. uncertainty about expectations or what to do
   5. Other; please specify*
4. What could the Next Gen PET FOLC do to make it more likely for you to be involved in a project group? [Display only if Q9= No or sort of/not sure]

**Other Ideas About Future NGP FOLC Activities**

1. What other Next Gen PET FOLC activities would you like to participate in? Check all that apply.

❏ Q&A with guest experts - one-time sessions either on video or ‘live’ on slack

❏ Scheduled discussions with someone external to the FOLC on specific topics

open to all FOLC members

❏ Community-wide webinars

❏ Video club - a group that meets regularly to share and reflect on videos of our

teaching

❏ “Taking it up a notch” groups that meet regularly to focus on improving a

particular aspect of our teaching (eg, whole class discussions, managing groups,

etc)

❏ A group that meets regularly to share and reflect on student work

❏ A working group to develop "implementer's perspective" guides for the

curriculum

❏ Peer feedback from the FOLC, possibly for use in teaching portfolio or tenure

materials

❏ Other; please specify*

❏ None of the above

1. Please share any ideas for additional FOLC activities.

**Your Thoughts About the Next Gen PET Curriculum**

1. Please respond to each of the following statements in terms of your present thinking about the Next Gen PET curriculum. Select one on each row. [0, Irrelevant;1, Not true of me now; 4, Somewhat true of me now; 7, Very true of me now]

a. I am concerned about how to organize my course using the Next Gen PET

curriculum.

b. I am concerned about not having enough time to organize myself each day to use

the Next Gen PET curriculum.

c. I am concerned about being able to acquire and manage the materials and

equipment the Next Gen PET curriculum requires.

d. I am concerned about my ability to implement the teaching strategies in the Next

Gen PET curriculum.

e. I am concerned about students’ attitudes towards the Next Gen PET curriculum.

f. I am concerned about students’ abilities to engage in the inquiry-oriented

activities included in the Next Gen PET curriculum.

g. I am concerned about how the Next Gen PET curriculum affects students’

learning.

h. I would like to help other faculty in their use of the Next Gen PET curriculum.

i. I would like to develop working relationships with other faculty using the Next

Gen PET curriculum.

j. I would like to coordinate my effort with others to maximize the effects of the

Next Gen PET curriculum.

k. I would like to know what other faculty are doing with the Next Gen PET

curriculum.

l. I now know of some other approaches that might work better than the Next Gen

PET curriculum.

m. I would like to revise the instructional approach of the Next Gen PET curriculum.

n. I would like to modify my use of the Next Gen PET curriculum based on the

experiences of my students.

o. I would like to determine how to supplement or enhance the Next Gen PET

curriculum.

1. Please indicate your current level of preparedness to do each of the following: [Not At All Prepared, Somewhat Prepared, Fairly Well Prepared, Very Well Prepared]
   1. Structure your course using the Next Gen PET curriculum
   2. Manage the equipment/logistics associated with implementing the Next Gen PET curriculum
   3. Teach the Next Gen PET curriculum materials effectively
   4. Assess student learning *formatively* in the context of the Next Gen PET curriculum
   5. Assess student learning *summatively* in the context of the Next Gen PET curriculum
2. To what extent has ***participating in the FOLC*** prepared you to do each of the following: [not at all, minimally, moderately, to a great extent]
   1. Structure your course using the Next Gen PET curriculum
   2. Manage the equipment/logistics associated with implementing the Next Gen PET curriculum
   3. Teach the Next Gen PET curriculum materials effectively
   4. Assess student learning *formatively* in the context of the Next Gen PET curriculum
   5. Assess student learning *summatively* in the context of the Next Gen PET curriculum

**Table S1. Participants’ Perceptions of Preparedness^†^ for Various Aspects of Implementing Next Gen PET**

|  | **Percent of Respondents**  **(N = 22)** | | |
| --- | --- | --- | --- |
|  | **Retrospective Pre-workshop** | **Post-**  **workshop** | **Follow-up** |
| Structure your course using the Next Gen PET curriculum | 30 | 87 | 100 |
| Manage the equipment/logistics associated with implementing the Next Gen PET curriculum | 43 | 78 | 100 |
| Teach the Next Gen PET curriculum materials effectively | 48 | 87 | 91 |
| Assess student learning summatively in the context of the Next Gen PET curriculum | 30 | 73 | 83 |
| Assess student learning formatively in the context of the Next Gen PET curriculum | 26 | 55 | 74 |

**^†^** Includes those respondents indicating 3 or 4 on a four-point scale from 1 “not at all prepared” to 4 “very well prepared.”

**Sense of Preparedness Composite Scores & the HLM Analysis**

The composite score for preparedness was calculated by summing the responses to the items in Figure 3 and then dividing by the total points possible. To put the composite on a 100-point scale, the lowest response option was set to 0, and the others were adjusted accordingly. The preparedness response scale, which originally ranged from 1 to 4, was recoded to have a scale of 0 to 3. The denominator was determined by computing the maximum possible sum of responses for the series of items. A respondent’s score was then calculated as (sum of responses/maximum possible sum)x100.

A two-level hierarchical linear model (HLM; Raudenbush & Bryk, 2002) was used to test whether there was a statistically significant difference over time on the composite scores. In the model, time points (level 1) were nested within participants (level 2).

Time point indicator variables were entered into the model uncentered; thus, the intercept represents the spring 2019 time point, and the coefficients estimated for the other time point variables test those time points against the spring 2019 time point.

Table S1 includes descriptive statistics on the raw scores for the composite by time point and overall.

**Table S2**

**Raw Composite Scores**

|  | **N** | **Minimum** | **Maximum** | **Mean** | **Std. Deviation** |
| --- | --- | --- | --- | --- | --- |
| Overall | 84 | 0 | 100 | 63.41 | 26.39 |
| Retrospective Pre-Workshop | 23 | 0 | 73 | 35.65 | 24.09 |
| Post-Workshop | 22 | 33 | 100 | 62.73 | 14.20 |
| Spring 2019 | 39 | 40 | 100 | 80.17 | 17.87 |

There was a significant difference in composite scores between the retrospective pre-workshop and spring 2019 time points, as well as between the post-workshop and the spring 2019 time points (HLM; p < 0.05). In terms of effect sizes (Cohen, 1988), scores in spring 2019 were 1.61 standard deviations higher than on the retrospective pre-workshop composite and 0.79 standard deviations higher than on the post-workshop survey composite.
